# Discovery of novel xylosides in co-culture of basidiomycetes *Trametes versicolor* and *Ganoderma applanatum* by integrated metabolomics and bioinformatics

**DOI:** 10.1038/srep33237

**Published:** 2016-09-12

**Authors:** Lu Yao, Li-Ping Zhu, Xiao-Yan Xu, Ling-Ling Tan, Martin Sadilek, Huan Fan, Bo Hu, Xiao-Ting Shen, Jie Yang, Bin Qiao, Song Yang

**Affiliations:** 1 School of Life Science, Qingdao Agricultural University, Shandong Province Key Laboratory of Applied Mycology, and Qingdao International Center on Microbes Utilizing Biogas, Qingdao 266109, Shandong Province, China; 2 Department of Chemistry, University of Washington, Seattle, Washington 98195, United States of America; 3 Tianjin Academy of Agricultural Sciences, Tianjin 300192, China; 4 Industrial Product Division, Intrexon Corporation, South San Francisco, CA 94080, United States of America; 5 Department of Biochemistry and Molecular Biology, School of Basic Medical Sciences, Tianjin Medical University, Tianjin 300070, China; 6 School of Chemical Engineering and Technology, Tianjin University, Tianjin 300070, China; 7 Key Laboratory of Systems Bioengineering, Ministry of Education, Tianjin University, Tianjin 300072, China

## Abstract

Transcriptomic analysis of cultured fungi suggests that many genes for secondary metabolite synthesis are presumably silent under standard laboratory condition. In order to investigate the expression of silent genes in symbiotic systems, 136 fungi-fungi symbiotic systems were built up by co-culturing seventeen basidiomycetes, among which the co-culture of *Trametes versicolor* and *Ganoderma applanatum* demonstrated the strongest coloration of confrontation zones. Metabolomics study of this co-culture discovered that sixty-two features were either newly synthesized or highly produced in the co-culture compared with individual cultures. Molecular network analysis highlighted a subnetwork including two novel xylosides (compounds **2** and **3**). Compound **2** was further identified as N-(4-methoxyphenyl)formamide 2-O-*β*-D-xyloside and was revealed to have the potential to enhance the cell viability of human immortalized bronchial epithelial cell line of Beas-2B. Moreover, bioinformatics and transcriptional analysis of *T. versicolor* revealed a potential candidate gene (GI: 636605689) encoding xylosyltransferases for xylosylation. Additionally, 3-phenyllactic acid and orsellinic acid were detected for the first time in *G. applanatum,* which may be ascribed to response against *T.versicolor* stress. In general, the described co-culture platform provides a powerful tool to discover novel metabolites and help gain insights into the mechanism of silent gene activation in fungal defense.

In natural ecosystem, interspecies interaction is a key feature of wood-decaying basidiomycetes. There are two major consequences of competitive interactions, one is named as replacement in which one basidiomycete gains the territory of another, and the other is called deadlock in which neither fungus gains advantages over the other^1^. It has been reported that competitive antagonism lead to various biochemical changes in the interaction zone of either replacement or deadlock, such as differential gene expression, up or down-regulation of extracellular enzymes, and diverse secondary metabolites production^2^,^3^. However, recent genome sequencing and transcriptomic analyses of basidiomycetes indicated that many gene clusters of pure fungi culture involved in metabolite synthesis were presumably silent or low expressed under standard laboratory cultivation,. Therefore, even though many kinds of secondary metabolites with medicinal actions of antitumor, immunomodulating, antioxidant, cardiovascular and antimicrobial have been isolated from basidiomycetes. there is still a large gap between discovered secondary metabolome and genome capability of basidiomycetes^4^.

The co-culture strategy mimics naturally occurring ecology by constructing an artificial microbial community. It has been widely applied to activate silent genes and reveal novel secondary metabolites in the bacteria, filamentous fungi and actinomycetes^5^,^6^,^7^. However, only a few researches about the co-cultivation of basidiomycetes have been reported, which mainly focused on studying secreted extracellular enzymes^8^,^9^,^10^,^11^. Our recent research found that several natural products, including one new sesquiterpenoid with an antiviral activity against H5N1 influenza A virus, were produced in mono-culture of basidiomycete *Phellinus ignarius* but at low yield^12^. Zheng *et al*. demonstrated that co-cultivation of the same genera *Phellinus punctatus* with another basidiomycete *Inonotus obliquus* resulted in lower production of mycelial biomass but an increased amount of metabolites^8^. Authors in reference^9^ showed that interacting mycelia of a wood-decaying basidiomycete, *Stereum hirsutum* with two competitor basidiomycetes, *Coprinus disseminates* and *C*. *micaceus*, up or down-regulated a number of metabolites in *S. hirsutum*, suggesting the potential of inducing dramatic physiological changes of microorganism by fungi-fungi co-culturing. In this research, we dedicated to develop a platform that enables systematical discovery of the novel metabolites in basidiomycetes during pairwise interspecies interactions.

The recent application of high-resolution mass spectrometry (MS)-based platforms has increased the number of detected metabolites but also faces several challenges. One challenge of MS-based analysis is low-throughput detection and characterization of metabolic features^13^. The MS data analysis tools such as MZmine 2 and XCMS have been developed to detect features, align samples and discover differences between sample groups^14^,^15^. Another challenge is inefficiency of dereplicating known compounds and their potential analogues. Molecular network analysis combined with tandem mass spectrometry has been recently developed as a promising dereplication strategy for secondary metabolites discovery in diverse bacterial genera^16^,^17^. Molecular networking allows for a simultaneous visual exploration of identical molecules, analogues, or compound families by comparing MS fragmentation similarity or common losses, thereby accelerating the process of structural resemblance^16^,^17^.

In this work, the interspecies mycelial interactions between fifteen wood-decaying basidiomycetes and two straw-decaying basidiomycetes were investigated on agar plates. Then the co-culture system of *T. versicolor* and *G. applanatum* showing a significant deadlock was chosen as an interactive model to discover the induced metabolites. We found that sixty-two features were either newly synthesized or significantly changed in the co-culture, and five of which were fully characterized by a combination of molecular networking, mass spectrometry and nuclear magnetic resonance spectroscopy. Two metabolites, identified as important carboxylic acids, were found to be significantly produced by *G.applanatum*, and two novel xylosides were abundantly synthesized by *T. versicolor*. Moreover, the candidate genes encoding for xylosyltransferase were identified in *T. versicolor*, and the biological activity of the novel xyloside on the two airway epithelial cell lines was further evaluated.

## Results

### Construction of the co-culture system between basidiomycetes

**Figure 1:**
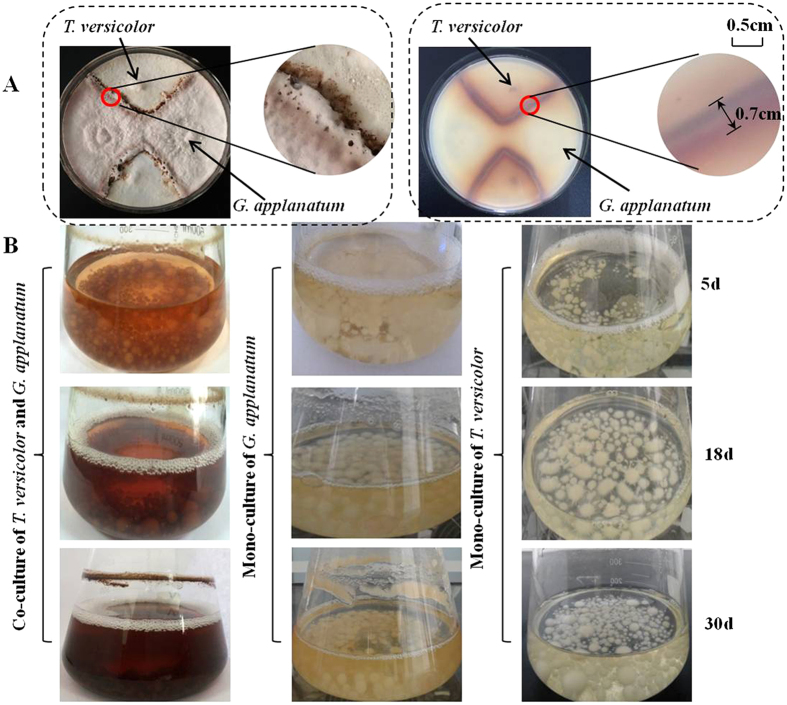
Co-culture of *T. versicolor* and *G. applanatum* on agar plate and in the flasks. (**A**) A pairwise co-culture of *T. versicolor* and *G. applanatum* on agar plate on day 18. (**B**) Comparison of co-culture (first column) with mono-cultures of *G. applanatum* (middle column) and *T. versicolor* (last column) in the flasks after 5, 18 and 30 days.

Fifteen wood-decaying basidiomycetes and two straw-decaying basidiomycetes were used to build up a pairwise co-culture system on agar plates as shown in Supplementary Table S1 and Fig. S1. These basidiomycetes were selected from different orders including Agaricales, Polyporales and Hymenochaetales. In total, 136 pairwise co-cultures were constructed and assessed, and 78 of them were able to form visible confrontation zone. For example, the co-culture of *T. versicolor* with *G.applanatum* or *T. robiniophia* with *Pleurotus nebrodensis* were able to develop a strong coloration of confrontation zones over 0.5 mm wide (Fig. 1A and Supplementary Fig. S1B). By contrary, the confrontation zone of the co-culture of *P. eryngii* with *G. sinense* was less than 0.2 mm wide (Supplementary Fig. S1A). The large size of confrontation zone usually represented significant metabolic activities, implying an appropriate model to explore novel metabolites^1^,^18^. Since *T. versicolor* and *G. applanatum* have been reported to possess a potential of synthesizing broad spectrum of secondary metabolites^19^,^20^,^21^, the co-culture of these two fungi was further assessed. As shown in Fig. 1B, the co-culture of *T. versicolor* and *G. applanatum* were inoculated in the flasks for 5 days to 30 days before sampling. In contrast to the unchanged color of mono-cultures, the color of co-cultured suspension transited from deep yellow on day 5 to dark red on day 18.

### Discovery of induced metabolites by data-driven processing

To discover metabolites induced by the interaction of *T. versicolor* and *G. applanatum*, the co-culture supernatant was extracted by a mixed organic solution (dichloromethane /methanol/water) and the extracts were injected into high resolution LC-MS system. The raw data was pre-processed by MZmine 2 and post-processed by an unsupervised principal component analysis (PCA) and heatmap analysis.

**Figure 2:**
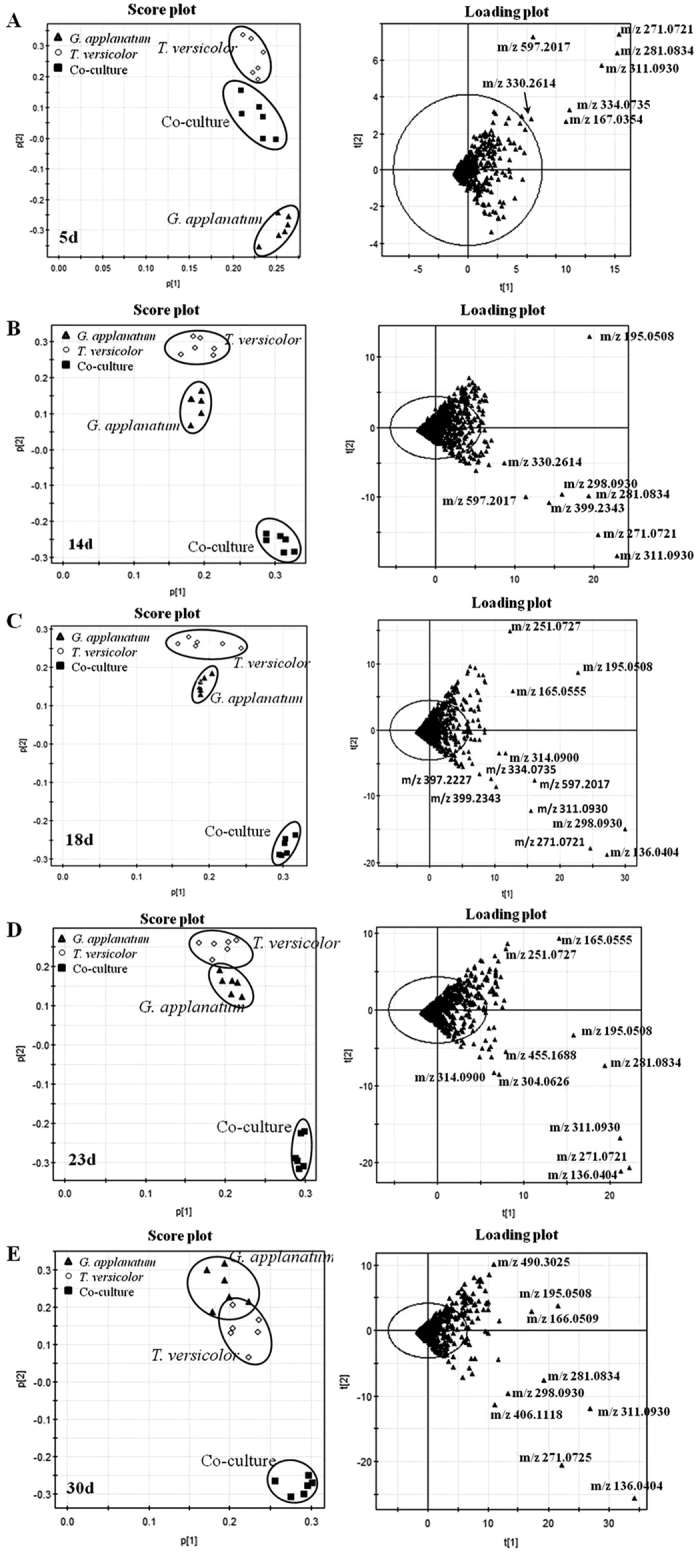
PCA of co-culture of *T. versicolor* and *G. applanatum* and their corresponding mono-cultures at different time points (5, 14, 18, 23 and 30 days). (**A** to **E**) The score and loading plots of the data analyzed by LC-MS in the negative mode. The scattered dots labeled with m/z were the representative features responsible for discriminating three samples. Data was acquired from three independent biological replicates and two analytical replicates.

A portion of the LC-MS chromatogram, containing the representative differential features, was shown in Supplementary Fig. S2. The data was acquired on day 23 for the co-cultures and corresponding mono-culture controls. Magnified extracted ion chromatograms suggested that features like m/z 298.0930, 300.1075 and 230.1155 were only synthesized in the co-culture, and the feature such as m/z 165.0555 was 5-fold higher in the co-culture than that in the mono-culture. PCA multi-statistical analysis was used to process and visualize the multi-dimensional metabolomic datasets (Fig. 2A to E). PCA provided clear separation among the three sample groups. The co-cultures were significantly different from two mono-culture controls at each time point, suggesting that some features only presented in co-cultures. The variables responsible for discriminating these samples were further demonstrated by loading plots. The labeled features were either only detected in the co-culture or their intensities were 5-fold higher than the mono-cultures on the same sampling day (Fig. 2A to E). Moreover, a heatmap analysis was applied to visualize and compare the datasets between the co-culture and their corresponding mono-cultures (Supplementary Fig. S3). Five features (i.e. m/z 140.0705, 181.0616, 200.0353, 334.0862 and 415.2261), which were not obtained by PCA, were further revealed by the heatmap analysis. Overall, fifty-seven features responsible for induction were successfully identified and thirty-eight of them were not detected at any time point in the mono-cultures, suggesting that fungal interactions were capable of activating or modulating the biosynthetic pathways to broaden the metabolite profiles (Supplementary Table S2).

### Dereplication of induced metabolites by molecular network analysis

**Figure 3:**
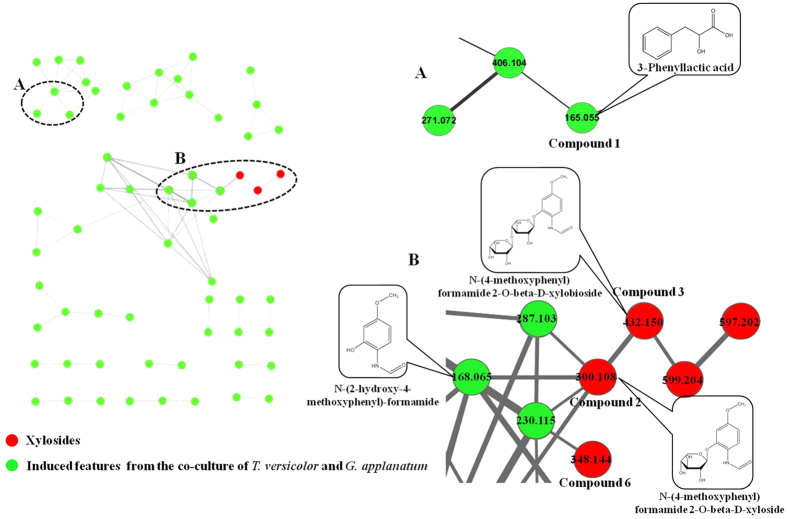
Molecular network analysis of induced features in the co-culture. (**A**) 3-Phenyllactic acid (m/z 165.0555) clustered to two potential carboxylic acids (m/z 406.1038 and 271.0725); (**B**) Potential novel xylosides (m/z 300.1075, 432.1502 and 348.1439) clustered to three other compounds of m/z 168.0655, 230.1155, 287.1029. The m/z 168.0655 was elucidated by NMR as N-(2-hydroxy-4-methoxyphenyl) formamide. The m/z 599.2043 was a dimmer of m/z 300.1075 and m/z 597.2017 was the negative ion of m/z 599.2043.

In order to clarify if induced features were metabolites that have been discovered, molecular networking of the MS/MS spectra were performed. Fragmental spectra were compared in pairwise accounting to fragmental masses and relative intensities. Subnetworks were generated only from induced features that did not match with database information (Fig. 3). For each subnetwork, nodes represent parent ions and edges represent their structural relationships. One example of dereplication was that molecular network analysis resulted in identifying 3-phenyllactic acid (m/z 165.0555, compound **1**) previously discovered by PCA (Figs 2 and 3A). This identification was further confirmed by comparing retention time, accurate mass and MS/MS fragments between the sample and the standard (Supplementary Figs S4A and S5A). 3-phenyllactic acid was clustered with other two induced features (m/z 271.0725 and 406.1038). The analysis of their MS/MS fragment ions indicated they possibly also belonged to the class of carboxylic acid (Supplementary Fig. S6A).

More interestingly, molecular network analysis highlighted a subnetwork composed of three glycosides and respective aglycone. As shown in Fig. 3B, molecules with m/z of 300.1075, 432.1502 and 348.1439 (compounds **2**, **3** and **6**) were found to cluster with another feature of m/z 168.0655. Comparison of their fragment ions shows a common neutral loss of 132 Dalton resulting from deglyosylation (Supplementary Fig. S6B). Moreover, for compound **2** and compound **3**, the residue mass after the loss of 132 produced very similar MS/MS pattern as compound **6** (Supplementary Fig. S6B), suggesting that both compounds were most likely synthesized by attaching one or two pentose moieties to compound **6**. In addition, other features of m/z 230.1155 and 287.1029 were clustered with m/z 168.0655 in the subnetwork. MS/MS fragments of these three ions indicated that they could possess similar structures as well (Supplementary Fig. S6C).

### Identification of induced metabolites

**Figure 4:**
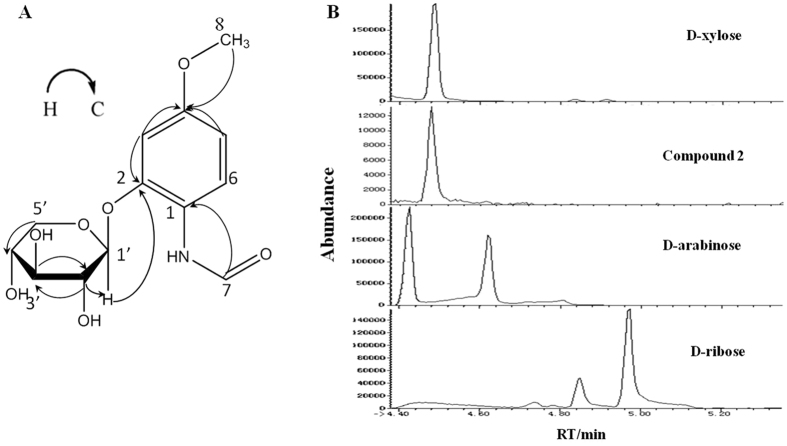
Identification of compound 2. (**A**) Key HMBC correlations of compound **2**. (**B**) Identification of glycone of compound **2** by GC-MS. Comparison of retention times between the product of acid hydrolysis of compound **2** and pentose standards (i.e. D-xylose, D-arabinose and D-ribose).

Feature of compound **2** was isolated as white powder and detected in high resolution LC-MS in the positive ion mode at m/z 300.1075, and thus C_13_H_17_NO_7_ was suggested as its putative molecular formula (calculated m/z 300.1078 for C_13_H_17_NO_7_, with an error of 1 ppm). The ^1^H and ^13^C NMR data of compound **2** showed that the presence of one pentosyl moiety, clearly indicated by one anomeric carbon signal at δ_C_ 104.40, and one anomeric proton signal at δ_H_ 4.81 (d, J = 7.8 Hz) (Supplementary Table S3). In addition, there was one hydroxymethyl signal at δ_H_ 3.77, one benzene signal at δ_H_ 6.62, 6.76, 8.04, suggesting that compound **2** possessed a 3-methoxyphenol skeleton as an aglycone. One more carbanyl signal at δ_H_ 8.27 was observed in the ^13^C NMR spectrum. Thus, the structure of aglycone (i.e. m/z 168.0655) of compound **2** was identified as N-(2-hydroxy-4-methoxyphenyl)formamide. In the HMBC spectrum, the long-range correlations between H-8 (δ_H_ 3.77) with C-4 (δ_C_ 159.0) suggested that hydroxymethyl group was substituted at the C-4 of compound **2** (Fig. 4A). Furthermore, the locations of the oxygen and nitrogen substituent mentioned above were confirmed to be at C-2, C-1, respectively, by the HMBC spectrum (Supplementary Fig. S8). To identify pentosyl moiety, compound **2** was acid-hydrolyzed and analyzed on GC-MS. According to retention time of standards and MS fragments, sugar moiety was confirmed to be D-xylose (Fig. 4B). Moreover, ^1^H NMR coupling constants (3 J_1,2_ > 7 Hz) were consistent with *β*-configuration for xylose. Finally, compound **2** was determined as a novel metabolite, N-(4-methoxyphenyl)formamide 2-O-*β*-D-xyloside, and compound **3** was determined as xylobioside, i.e. N-(4-methoxyphenyl)formamide 2-O-*β*-D-xylobioside (Table 1). To identify other features, each corresponding MS/MS spectra was blasted against several public databases including METLIN, MassBank and ReSpect. Features of m/z 195.0508 and 167.0354 (compounds **4** and **5**) were further identified as gluconic acid and orsellinic acid, and they were confirmed by comparison with the standards (Supplementary Figs S4 and S5).

Other xylosides in co-cultured supernatant were also detected because the xylosylation contribute to the increase in water solubility of aglycone. Five other xylosides with m/z 242.1024, 270.0979, 272.1128, 369.2269 and 400.1019 (compounds **7** to **11**) were further discovered by neutral loss scanning for a loss of 132 Dalton and confirmed by MS/MS scan (Table 1 and Supplementary Fig. S7). These xylysoides were also only detected in the co-culture (Supplementary Table S2). As a summary, a total of sixty-two induced features were successfully identified in this study.

### Dynamics of induced features accumulated in fungal co-culture

The induced features were quantified over time in order to determine the tendency of production, which suggests that many features were up-regulated along with the time. For example, the compounds **2** and **3** were respectively detected on day 5 and 18, and their intensities kept increasing till day 30 (Supplementary Fig. S9A). Moreover, some features accumulated to the maximum abundance at the early days but decreased afterwards, such as compound **1** (m/z 165.0555), compound **5** (m/z 167.0354), and compound **6** (m/z 348.1439) (Supplementary Fig. S9B). Compound **1** showed the maximum abundance on day 14, which was 14-fold higher than that in the mono-culture of *G. applanatum*. And the abundance of compound **5** was highest on the day 5, which was 12-fold higher than *G. applanatum*. The time-dependent changes of all the features were summarized in Supplementary Fig. S9.

### Co-cultivation stimulates *T. versicolor* and *G. applanatum* produce different metabolites

**Figure 5:**
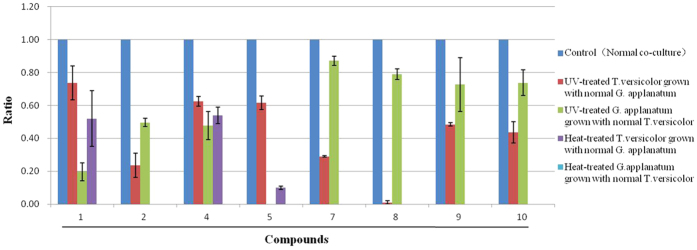
Comparison of the abundance of compounds between treated fungi and the control. . The y axis is the ratio of the compounds abundance of UV/heat-treated fungi co-culture to the normal co-culture (the control). The average value for the control was set to 1. Data show the mean with error bars indicating standard deviation calculated from three independent biological replicates.

In order to associate the induced metabolites with corresponding production fungi, we first treated the fungal cultivation with the heat-killed mycelia. All newly features were not observed in either *T. versicolor* or *G. applanatum*. Furthermore, *T. versicolor* and *G.applanatum* were exposed under an ultraviolet light to decrease the growth activity (Supplementary Fig. S10). As shown in Fig. 5, when low active mycelia of *G. applanatum* was co-cultivated with *T. versicolor*, the abundances of compound **2** and other four xylosides (compounds **7** to **10**) were 2.01, 1.15, 1.27, 1.37, 1.35-fold lower compare to the normal co-culture. On the contrary, when low active mycelia of *T. versicolor* were co-cultivated with *G. applanatum,* these xylosides were either hardly detected (compound **8**) or 2.07 to 6.67 lower than the normal co-culture (compound **2**, **7**, **9**, **10**). This test proved that the synthesis of xylosides was most likely activated in *T. versicolor* in the presence of *G. applanatum*. Moreover, the abundances of 3-phenyllactic acid and orsellinic acid (compounds **1** and **5**) were 1.36 and 1.63-fold lower than the normal co-culture when low active mycelia of *T. versicolor* was added to *G. applanatum*, but they were highly reduced when low active mycelia of *G. applanatum* was added to *T. versicolor* (Fig. 5), suggesting that the accumulation of 3-phenyllactic acid and orsellinic acid in the co-culture were mainly result from the activation of *G. applanatum.* In addition, xylosides of compounds **3**, **6** and **11** were not able to be detected in both conditions, suggesting that synthesis of those compounds requires interacted live mycelia or presence of induced signal molecules.

### Bioinformatics predicts possible xylosyltransferase and real-time quantitative PCR analysis in *T. versicolor*

**Figure 6:**
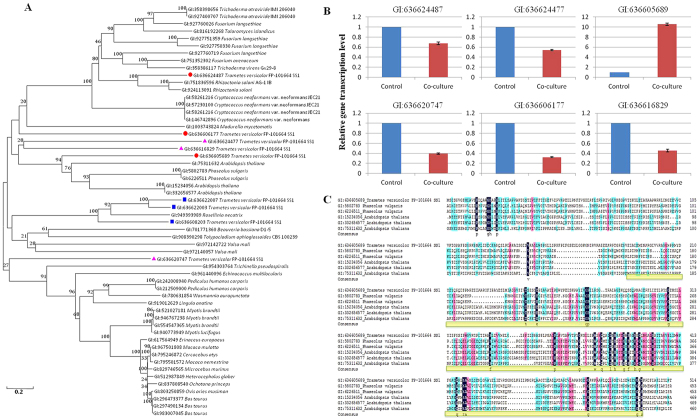
Bioinformatics predicts possible xylosyltransferase in *T. versicolor.* (**A**) Phylogenetic diversity of the potential xylosyltransferases from *T. versicolor* and the objective ones from NCBI database. Predicted *T. versicolor* xylosyltransferases are highlight in color symbols. Red circle means very likely; pink triangle means likely; Blue square means less likely. Numbers within the tree indicate the occurrence (%) of the branching order 1,000 bootstrapped trees, with a value range between 20 and 100; (**B**) Comparison of the transcriptional levels of predicted xylosyltransferases between the co-culture and mono-culture of *T. versicolor*. The average value for the control (i.e. mono-culture of *T. versicolor*) was set to 1. Data show the mean with error bars indicating standard deviation calculated from three independent biological replicates. (**C**) Conserved sequence analysis of potential xylosyltransferases (GI:636605689) in *T. versicolor*. Conservation of residues is denoted by colors: amino acids on dark blue background are strictly conserved; amino acids on pink background are high in similarity, while those on light blue are over than 50% of similarity. Yellow line indicates glycosyltransferase_GTB_type superfamily.

In order to gain insights into possible xylosyltransferase in *T. versicolor*, we blasted the proteome of *T. versicolor* against all the xylosyltransferase and related proteins in NCBI database. With the cutoff value settled at query length over 200 bp and identity over 25%, nine most significant hits from *T. versicolor* and forty-six significant blast hits from NCBI database were screened out (Supplementary Table S4). A phylogenetic analysis of fifty-four protein sequences revealed that GI:636624487, GI:636605689, GI:636606177, GI:636624477, GI:636620747 and GI:636616829 were possible xylosyltransferase in *T. versicolor* (Fig. 6A). To further investigate these predictions, gene transcriptional levels between the co-culture and mono-culture of *T. versicolor* were compared and shown in Fig. 6B. The transcriptional levels of GI:636624487, GI:636606177, GI:636624477, GI:636620747 and GI:636616829 were down-regulated in the co-culture compared to the mono-culture. In contrast, GI:636605689 gene was highly transcribed to be 10.5-fold higher in the co-culture compared to the mono-culture. A multiple-sequence alignment was further made between predicted xylosyltransferase (GI:636605689) and known xylosyltransferase to compare important conserved regions (Fig. 6C). The regions for glycosylation were shown as glycosyltransferase_GTB_type superfamily with high similar among the whole cohort.

### Biological activity of compound 2

**Figure 7:**
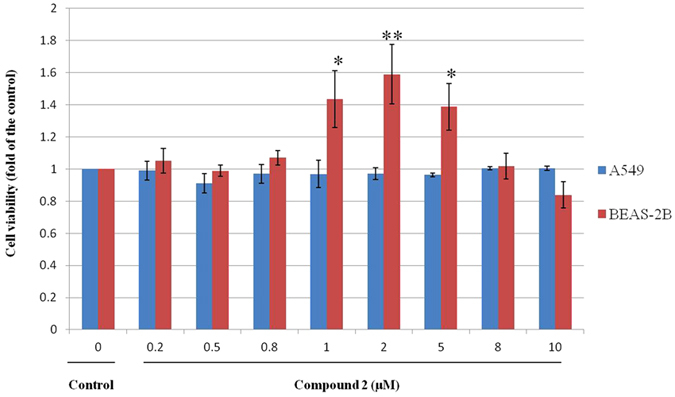
Addition of compound 2 affects the cell viability of BEAS-2B. The Y axis was the ratio of cell viability of compound **2-**treated samples to the control sample. Data show the mean with error bars indicating standard deviation calculated from at least three independent biological replicates. * and ** mean that the significance of cell viability between the treated samples and control sample was determined by t-tests (Origin 8.0) with a *p*-value less than 0.05 and 0.01 considered to be statistically significant.

To demonstrate the biological activity of compound **2**, two different airway epithelial cell lines (A549 and Beas-2B) derived from the human respiratory tract were treated with compound **2** at various concentrations for 48 h. As shown in Fig. 7, no visible changes of cell viability were observed for human lung cancer cell line A549 at any concentrations of compound **2**, suggesting it has no biological function for A549 cell. However, an interesting tendency was observed for human immortalized bronchial epithelial cell line BEAS-2B. The cell viability was increased by about 1.4-fold with the addition of 1 to 5 μM compound **2**, but was reduced back to the normal level when the concentration was increased to 10 μM. It implied that compound **2** has potential to enhance the cell viability of Beas-2B at relatively low concentrations.

## Discussion

In this work, we aimed to systematically study novel metabolites synthesized in basidiomycetes by establishing an artificial symbiotic platform mimicking fungal interactions in nature. We first studied the co-cultivation of seventeen basidiomycetes, and then focused on the co-cultivation of *T. versicolor* with *G. applanatum*, which displayed significant deadlock among 136 pairs. Comparison of secondary metabolite profiles of the extracts from mono-cultures of *T. versicolor* and *G. applanatum* and from their co-culture revealed sixty-two features were produced by the induced fungus. The induction patterns of some features were time-dependent, suggesting that relevant genes activation and their transcription and translation may occur at different growth status^22^. This result also highlighted the complexity of metabolomic analysis of basidiomycete interactions. Most interestingly, a series of newly xylosides and increased carboxylic acids were accumulated in the co-culture and found to be generated by *T. versicolor* and *G. applanatum* respectively, suggesting the different response mechanism of basidiomycete chemical defenses.

Glycosylation is one of the most important post-modification processes for secondary metabolites. From the perspective of chemical defense, the glycosylation of cytotoxic metabolites increase their solubility, which can help increase resistance against biotic or abiotic stress^23^. For instance, high concentrations of geraniol have been shown to be phytotoxic to maize, and temporary storage as glycoside and subsequent stress-induced enzymatic release of geraniol from its glycosides was used in maize as a primary defense strategy^24^. Moreover, glycoside can also possess strong bioactivity by themselves, for example constitutive accumulation of a resveratrol glucoside in transgenic alfalfa increased resistance to fungal pathogen *Phomamedic aginis*^25^. In addition, xylosyltransferase was found to be critical for plant development and growth under low and high temperatures, salt, drought and oxygen deficiency conditions^26^. In this work, we demonstrated that novel phenolic aglycones (compounds **2** and **3)** and other metabolites (compounds **6** to **11**) were xylosylated in *T. versicolor* during the co-culturing with *G. applanatum*. Previously Kondo *et al*. discovered that when 4-methylguaiacol was added to a culture inoculated with *T. versicolor*, it was xylosylated to 2-methoxy-4-methylphenyl *β*-D-xyloside^27^. Hundt *et al*. also found that *T. versicolor* had an ability to metabolize an antimicrobial compound triclosan to xyloside, and demonstrated that the formation of xyloside was attributed to the action of a xylosyltransferase^28^. We blasted the proteome of *T. versicolor*, and found that six potential proteins might respond to xylosylation. Among them, the predicted xylosyltransferase (GI:636605689) was highly expressed in the co-culture, suggesting it could be the major function for xylosylation. Furthermore, different types of xyloside were produced in *T. versicolor*, suggesting that the activated glycosyltransferase might be a promiscuous enzyme that is not only able to convert phenolic compounds but also other aglycones to corresponding xylosides. Recently, Ohgami *et al*. identified a UDP-glycosyltransferase from *Camellia sinensis*, and demonstrated its broad substrate specificity for sugar acceptors supported the structural diversity of the *β*-primeverosides of monoterpenes and primary alcohols in the tea leaves^29^. In the same report, however, another purified glycosyltransferase only specifically catalyzed the xylosylation of aroma glucosides^29^. Therefore, kinetic study of glycosyltransferase in *T. versicolor* is required to clarify whether single or multiple enzymes were involved in the xylosylation and their roles in fungal defenses.

Several glycosides structurally similar with compound 2 are high-value medicals, such as salidroside and gastrodin, so the medicinal properties of compound **2** were further studied^30^,^31^. In antibacterial assay, compound **2** had slight inhibition on *Cryptococcus neoformans* (ATCC 90012), *Bacillus subtilis* (ATCC 9372) and *Streptococcus sp.* (Supplementary Fig. S11), and had no influence on *Candida albicans* and gram-negative bacteria such as *Escherichia coli*. Most interestingly, compound **2** at micromole concentration had an ability to enhance the cell viability for a human immortalized bronchial epithelial cell line Beas-2B, but not for a human lung cancer cell line A549. In recent years, air pollution, typified by higher levels of PM2.5, has become a great concern for the public health in Asian countries such as China and India. PM2.5 adsorbed more harmful substances including metals and polycyclic aromatic hydrocarbons, which can be delivered easily through the respiratory tract inducing the damage of airway epithelial cell^32^,^33^. Thus, compound **2** may have the potential to be developed as a lead drug to relieve the injury of airway epithelial cell caused by external environment or cigarette smoke^34^. By contrast, due to the low production level of compound **3** and other xylosides (compounds **6** to **11**), their biological activities did not evaluate further. Additional isolation and purification are required to clarify the biological function of these xylosides.

Three carboxylic acids, 3-phenyllactic acid, gluconic acid and orsellinic acid (compounds **1**, **4** and **5**) were highly up-regulated in the co-culture, presumably due to the activation of *G. applanatum* with the interaction of *T. versicolor.* 3-phenyllactic acid has been reported as an antimicrobial compound against a broad range of microbes including *Listeria monocytogenes*, *S. aureus*, *E. coli*, yeasts, *Aspergillus ochraceus* and *Penicillium roqueforti*^35^. Orsellinic acid is a widespread building block for polyketides in fungi, and *OrsA* from *A. nidulans* and *ArmB* from *Armillaria mellea* have been described as fungal orsellinic acid synthases^36^,^37^. Moreover, it has been reported that orsellinic acid synthases was specifically activated to enhance the production of orsellinic acid when *A. nidulans* was co-cultured with *Streptomyces hygroscopicus*^5^. Gluconic acid was known as an oxidation product of glucose, and the synthesis of gluconic acid has been suggested to reduce environmental pH values, which might enhance its competence with other microorganisms^38^. In this study, the production of 3-phenyllactic acid and orsellinic acid by *G. applanatum* were significantly increased during the co-cultivation, suggesting that their encoding genes or gene clusters were activated to respond to *T. versicolor* and relieve its biotic stress in the co-culture. As far as we know, it was the first time to report that 3-phenyllactic acid and orsellinic acid were inducibly produced in *Ganoderma* genera. These findings indicated that the basidiomycetes of same order can active defense response in a different way, which open brand new perspectives in natural products research in the future.

## Conclusions

In this work, co-cultivation of *T. versicolor* and *G. applanatum* was developed as an interactive model for activating the silent genes to obtain novel metabolites. Data-driven approach discovered sixty-two features, which were either newly synthesized or highly produced in the different induction pattern over time of the co-culturing process. Among them, a series of xylosides were found to be highly synthesized in *T. versicolor* co-cultured with *G. applanatum*. Two of them (compounds **2** and **3**) were identified as novel compounds, N-(4-methoxyphenyl)formamide 2-O-*β*-D-xyloside and N-(4-methoxyphenyl)formamide 2-O-*β*-D-xylobioside. Biological activity assay demonstrated that low level of compound **2** has potential to enhance the cell viability of a human immortalized bronchial epithelial cell line Beas-2B, suggesting that it could be designed as a lead medicine for relieving the injury of airway epithelial cell. Moreover, a bioinformatics analysis of the whole proteome of *T. versicolor* predicted six possible genes encoding xylosyltransferases response for xylosylation, and real-time quantitative PCR analysis confirmed that GI:636605689 gene was likely appropriate candidate. This gene and its encoding enzyme can be focused on investigating the substrate specificity and reaction selectivity to identify the xylosylated function in the future. Additionally, two important carboxylic acids, 3-phenyllactic acid and orsellinic acid (compounds **1** and **5**) were found out to be significantly generated by *G. applanatum* for the first time. Overall, the collected information from current research provides a valuable platform to discover other novel secondary metabolites, and will be useful to uncover the mechanism of silent gene activation and novel gene function of basidiomycetes in the future.

## Materials and Methods

### Reagents

All chemicals including metabolite standards (3-phenyllactic acid, gluconic acid, orsellinic acid, D-xylose, D-ribose and D-arabinose) were purchased from either Sigma-Aldrich (St. Louis, MO, USA) or TGI (Kita-ku, Tokyo, JAPAN). Millipore water (Billerica, MA, USA) was used for the preparation of all the media, standards, and sample solutions.

### Fungus material and culture conditions

Seventeen basidiomycetes (i.e. *Pleurotus ostreatus, Lentinus edodes, Agaricus bisporus, Flammulina velutipes, Grifola frondosa* CGMCC No.8117*, Pleurotus eryngii, Ganoderma applanatum* CGMCC No.*5.249, Fructificatio Amaurodermatis Rudae, Ganoderma lucidum, Pleurotus nebrodensis, Phellinus igniarius* CGMCC No.8108*, Cordyceps militaris, Boletus edulis, Trametes versicolor* CGMCC No.12241*, Trametes robiniophia, Schizophyllum commune, Sclerotium Xylariae Nigripis*) were deposited at the Shandong Province Key Lab of Applied Mycology, China. The fungus was identified based on analysis of internal transcribed spacer sequences^39^,^40^. The culture medium consisted of glucose (2 g), KH_2_PO_4_ (0.2 g), MgSO_4_ (0.1 g), and peptone (0.4 g), and agar (4 g, only in solid medium) in 200 mL of sterilized water.

### Co-culture of basidiomycetes on agar plate

The co-culture procedure was adapted from the previous publication^22^, and co-culture medium was the same as above. Briefly, a 5 mm agar plug of each fungus was pre-cultured on a Petri dish (9 cm diameter), and were incubated at 28 °C. Co-culture experiments were inoculated with two 3 mm agar plugs of the fungal strains on opposite sides of an agar plate and incubated at 28 °C up to 30 days.

### Co-culture of *T. versicolor* and *G. applanatum*in in liquid medium and sample extraction

Five 3 mm agar plugs of *T. versicolor* and *G. applanatum* were pre-cultured in the 500 mL shake flask for 3 to 4 days. Then 50 mL of *T. versicolor* was transferred into the culture of *G. applanatum* and 50 mL of fresh medium was added. *T. versicolor* and *G. applanatum* were co-cultivated at 28 °C up to 30 days on orbital shakers at 180 rpm.

At the harvest time, 10 mL of culture broth was filtered and the filtrate was dried in a Free-dryer ALPHA 1-2 LDplus (Christ, Osterode, Germany). 5 mL of freshly prepared dichloromethane/methanol/water (64:36:8, v/v) solvent mixture was added to the dried samples^6^,^41^. The extractions were performed in a water bath sonicator (KQ-300GVDV, Kunshan, China) at room temperature for 20 min, and were centrifuged at 12000 rpm for 10 min. Finally, the extracts were redried in a Rotational Vacuum Concentrator (Christ, Osterode, Germany) and stored at −80 °C.

### Measurement of the metabolome

The extracts were dissolved in 200 μL methanol, and then were centrifuged at 12000 rpm for 10 min. The supernatants were transferred into 250 μL of Agilent autosampler vials. The sample was analyzed on an Agilent LC-QTOF (Agilent 1290 Infinity-6530B, Agilent Technologies, Santa Clara, CA, USA) as the previous publications^42^,^43^. Briefly, the m/z range was set to 50–1200 in centroid mode with a scan rate of 1.5 spectra/s. The sample was separated on an Acquity UPLC BEH C18 column (100 × 2.1 mm, 1.7 μm; Waters, Milford, MA, USA). The mobile phase A was water with 0.1% formic acid. The mobile phase B was pure acetonitrile. The linear gradient was as following: 0–4 min, 5% B; 4–5 min, 5–60% B; 5–11 min, 60–77.5% B; 11–21 min, 77.5–95% B; 21–24 min, 95% B; 24–24.5 min, 95–5% B; 24.5–30 min, 5% B. The injection volume was 3 μL.

### Data pre-processing and statistical analysis

LC-QTOF data were converted into mzML format using MS Convert software^44^. Data pre-processing and statistical analysis were performed with MZmine 2 (Version 2.11), SIMCA-P 11.5, and Metaboanalyst 3.0^14^,^45^. All the samples had three independent biological replicates and two analytical replicates. The significance was determined by t-tests (Origin 8.0) with a *p*-value less than 0.05 considered to be statistically significant.

### Molecular network analysis

MS/MS Data for molecular network analysis were acquired in targeted MS/MS on the same system of LC-QTOF. The collision energy and m/z range of different parent ions were optimized in terms of their own characteristics. MS/MS data was converted to mzML format, and then were subjected to the Molecular Networking workflow of Global Natural Products Social Molecular (GNPS at gnps.ucsd.edu) using the Group Mapping feature^16^,^17^. The following settings were used for the generation of the network: Minimum pairs cosine 0.5, parent mass tolerance 1.0 Dalton, ion tolerance 0.3 Dalton, maximum connected components 50, minimum matched peaks 6, minimum cluster size 2. The networks were visualized in the software program Cytoscape (Version 2.8.2)^46^.

### Isolation and purification of compound 2

The mycelia in the culture broth (15 L) were filtered and the filtrate was extracted three times with Ethyl acetate (EtOAc). The crude EtOAc extract (1.1 g) was concentrated under reduced pressure and then purified on a silica gel (200–300 mesh) column, eluting stepwise with petroleum ether/ethyl acetate and chloroform/methanol system to yield eight fractions^12^. Fraction 5 (93 mg) from the chloroform/methanol elution (10:1, v/v) was applied to a Medium Pressure Liquid Chromatography (Flash CO140080-0, Agela Technologies, China) and eluted with methanol/water (20–50% MeOH in 40 min) to yield a mixture (30 mg). This mixture was purified on a silica gel (200–300 mesh, Qingdao Haiyang Chem. Ind. Co. Ltd. China) column by eluting with chloroform/methanol (30:1, v/v) and then separated on a preparation column (Venusil XBP C18 (2), Agela Technologies, China) by eluting with methanol/water (5–30% MeOH in 40 min, 8 mL/min) to yield compound **2** (7.5 mg).

### NMR analysis of compound 2

1 H, 13 C and 2D NMR spectra of the purified compound **2** were recorded on a Brucker Avance 600 MHz instrument at 25 °C, using deuterated methanol as the internal lock. The resulting spectra were manually phased and baseline corrected, and calibrated to methanol, using TOPSPIN (version 2.1, Bruker).

### Acid hydrolysis and determination of the glycone of compound 2

Compound **2** (0.5 mg) was hydrolyzed with 2 M trifluoroacetic acid (1 mL) at 120 °C for 1 h, then hydroxylamine hydrochloride (10 mg) was added. The reaction mixtures were acetylated with acetic anhydride/pyridine (1:1, 1.0 mL) at 120 °C for 30 min and analyzed on an Agilent 5975B/6890 N GC-MS instrument (Agilent Technologies, Santa Clara, CA, USA). The sample was separated on a Thermo TR-5 MS column (60 m × 0.25 mm × 2.5 μm)^47^. The glycone of compound **2** was determined by comparing the retention time and MS/MS spectra with the sugar standards.

### Neutral loss scanning of the samples in water phase

10 mL of cultured supernatant were directly dried down to 0.5 mL and then were filtered using Nylon Syringe Filters (0.22 μm, 13 mm). Neutral loss experiments were carried out on an Agilent LC-QQQ-MS system (Agilent 1290 Infinity-6460, Agilent Technologies, Santa Clara, CA, USA) in both positive and negative modes. The mobile phase and gradient were as the same as LC-QTOF analysis. Each neutral loss spectrum was scanned from m/z 150 to 1000 in centroid mode with a scan rate of 1.5 spectra/s.

### Sequence and Phylogenetic analysis

To search for xylosyltransferase genes, all amino acid sequences of *T. versicolor* were applied as queries in a blast search against all xylosyltransferase relative proteins from NCBI protein database. Proteins were considered as possible xylosyltransferases if the query length of the blast hits was over 200 bp and identity score over 25%, respectively. All xylosyltransferases candidates together with their blast hits were further imported into MEGA 6.06 for phylogenetic analysis and construction of trees^48^. Sequences were aligned with ClustalW with MEGA 6.06 default parameters and the irregular sequences at the two ends were cut. The evolutionary history was calculated using the Neighbor-Joining method. Bootstrap tests were performed with 1000 replicates. The evolutionary distances were computed using the Poisson correction method and are in the units of the number of amino acid substitutions per site. All positions with less than 95% site coverage were eliminated and then output the phylogenetic tree. In addition, multiple-sequence alignment was performed using DNAMAN v 6.0.3.99 subsequently to generate the conservation properties of the predicted xylosyltransferases.

### RNA extraction and real-time quantitative PCR (qPCR)

On day 18 of liquid fermentation, the mycelia of *T. versicolor* in the co-culture and mono-culture, were harvested and stored at −80 °C, respectively. The mycelia were ground to powder under liquid N_2_. Total RNA was extracted according to standard protocols of the UNlQ-10 column Trizol Total RNA Extraction Kit (Sangon, China) and cDNAs was obtained using the AMV First Strand cDNA Synthesis Kit (NEB, USA). The single stranded cDNA was 1/10 diluted for qPCR analysis using the primer pairs (Supplementary Table S5) and 2X SG Fast qPCR Master Mix (High Rox) (Sangon, China) for each of the six candidate genes. GAPDH was employed as a reference gene^49^. qPCR was performed in accordance with the manufacturer’s instructions. Each reaction was conducted in biological triplicate. The Ct values obtained were used as the original data to calculate the relative transcriptional expression level of the candidate genes normalized against that of GAPDH gene. In correlation analysis, we normalized the genes expression levels by the 2^−△△Ct^ method^50^. The qRT-PCR product was also identified by 2% agarose gel electrophoresis.

### Cell viability assay

The human immortalized bronchial epithelial cell line BEAS-2B and human lung cancer cell line A549 were obtained from American Type Culture Collection (Manassas, VA, USA). They were cultured in DMEM (HyClone, USA) containing 10% FBS (fetal bovine serum, Gibco) and in RPMI-1640 (HyClone, USA) with 10% FBS, respectively^51^. The effect of compound **2** on the viability of BEAS-2B and A549 cells was assessed with CellTiter 96^®^ AQueous One Solution (Promega, Madison, WI, USA)^52^. Cells were seeded in 96-well plates at a density of 3000 cells per well overnight and subjected to different concentrations of compound **2** for 48 h including ethanol (culture medium). CellTiter 96^®^ AQueous One Solution reagent was then added to each well and incubated for 4 h according to manufacturer’s instructions. The absorbance was read at 490 nm with a microplate spectrophotometer (Multiskan FC, Thermo scientific, USA). Three independent experiments were performed with 6 replicates each and the results were expressed as a fold change relative to the control sample (i.e. no compound **2** added).

## Additional Information

**How to cite this article**: Yao, L. *et al*. Discovery of novel xylosides in co-culture of basidiomycetes *Trametes versicolor* and *Ganoderma applanatum* by integrated metabolomics and bioinformatics. *Sci. Rep.* **6**, 33237; doi: 10.1038/srep33237 (2016).

## Supplementary Material

Supplementary Information

## Figures and Tables

**Table 1:** List of the identified compounds and potential xylosides in the co-culture of *T. versicolor* and *G. applanatum.*

Name	Compounds	Predictive Elemental Composition	m/z	Difference	Classification
3-Phenyllactic acid	**1**	C_9_H_10_O_3_	165.0555[M−H]^−^	increased	Carboxylic acids
N-(4-methoxyphenyl)formamide 2-O-beta-D-xyloside	**2**	C_13_H_17_NO_7_	298.0930[M−H]^−^ 300.1075[M+H]^+^	newly synthesized	Glycosides
N-(4-methoxyphenyl)formamide 2-O-beta-D-xylobioside	**3**	C_18_H_25_NO_11_	432.1502[M+H]^+^	newly synthesized	Glycosides
Gluconic acid	**4**	C_6_H_12_O_7_	195.0508[M−H]^−^	increased	Carboxylic acids
Orsellinic acid	**5**	C_8_H_8_O_4_	167.0354[M−H]^−^	increased	Carboxylic acids
Undetermined	**6**	C_18_H_21_NO_6_	348.1439[M+H]^+^	newly synthesized	Glycosides
Undetermined	**7**	C_11_H_15_NO_5_	242.1024[M+H]^+^	newly synthesized	Glycosides
Undetermined	**8**	C_12_H_15_NO_6_	270.0979[M+H]^+^	newly synthesized	Glycosides
Undetermined	**9**	C_12_H_17_NO_6_	272.1128[M+H]^+^	newly synthesized	Glycosides
Undetermined	**10**	C_20_H_32_O_6_	369.2269[M+H]^+^	newly synthesized	Glycosides
Undetermined	**11**	C_20_H_17_NO_8_	400.1019[M+H]^+^	newly synthesized	Glycosides
