# Supplementary material for: Discovery of novel xylosides in co-culture of basidiomycetes Trametes versicolor and Ganoderma applanatum by integrated metabolomics and bioinformatics
Source: Sci Rep. 2016 Sep 12;6:33237. doi: 10.1038/srep33237 (PMC5018966; doi:10.1038/srep33237)
Supplement: Supplementary Information [file srep33237-s1.pdf]

Supplementary Information for:

Discovery of novel xylosides in co-culture of basidiomycetes *Trametes versicolor* and *Ganoderma applanatum* by integrated metabolomics and bioinformatics

Lu Yao[1], Li-Ping Zhu[1], Xiao-Yan Xu[1], Ling-Ling Tan[1], Martin Sadilek[2], Huan Fan[3], Bo Hu[4], Xiao-Ting Shen[1], Jie Yang[5], Bin Qiao[6], Song Yang[1, 7,*]

1. School of Life Science, Qingdao Agricultural University, Shandong Province Key Laboratory of Applied Mycology, and Qingdao International Center on Microbes Utilizing Biogas, Qingdao266109, Shandong Province, China
2. Department of Chemistry, University of Washington, Seattle, Washington 98195, United States of America
3. Tianjin Academy of Agricultural Sciences, Tianjin 300192, China
4. Industrial Product Division, Intrexon Corporation, South San Francisco, CA 94080, United States of America
5. Department of Biochemistry and Molecular Biology, School of Basic Medical Sciences, Tianjin Medical University, Tianjin 300070, China
6. School of Chemical Engineering and Technology, Tianjin University, Tianjin 300070, China
7. Key Laboratory of Systems Bioengineering, Ministry of Education, Tianjin University, Tianjin 300072, China

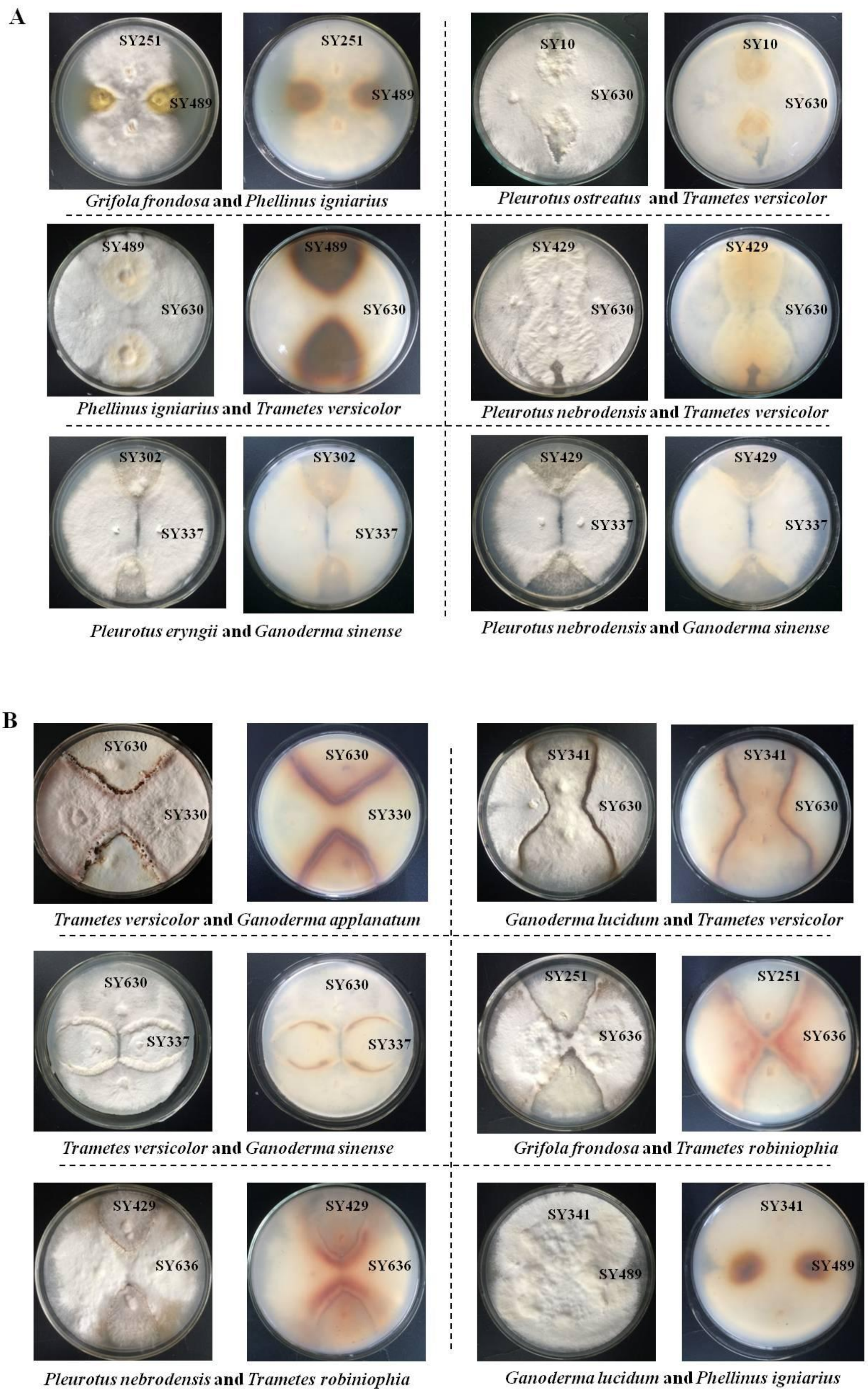
A
SY251
SY489
Grifola frondosa and Phellinus igniarius
SY10
SY630
Pleurotus ostreatus and Trametes versicolor
SY489
SY630
Phellinus igniarius and Trametes versicolor
SY429
SY630
Pleurotus nebrodensis and Trametes versicolor
SY302
SY337
Pleurotus eryngii and Ganoderma sinense
SY429
SY337
Pleurotus nebrodensis and Ganoderma sinense
B
SY630
SY330
Trametes versicolor and Ganoderma applanatum
SY341
SY630
Ganoderma lucidum and Trametes versicolor
SY630
SY337
Trametes versicolor and Ganoderma sinense
SY251
SY636
Grifola frondosa and Trametes robiniophia
SY429
SY636
Pleurotus nebrodensis and Trametes robiniophia
SY341
SY489
Ganoderma lucidum and Phellinus igniarius

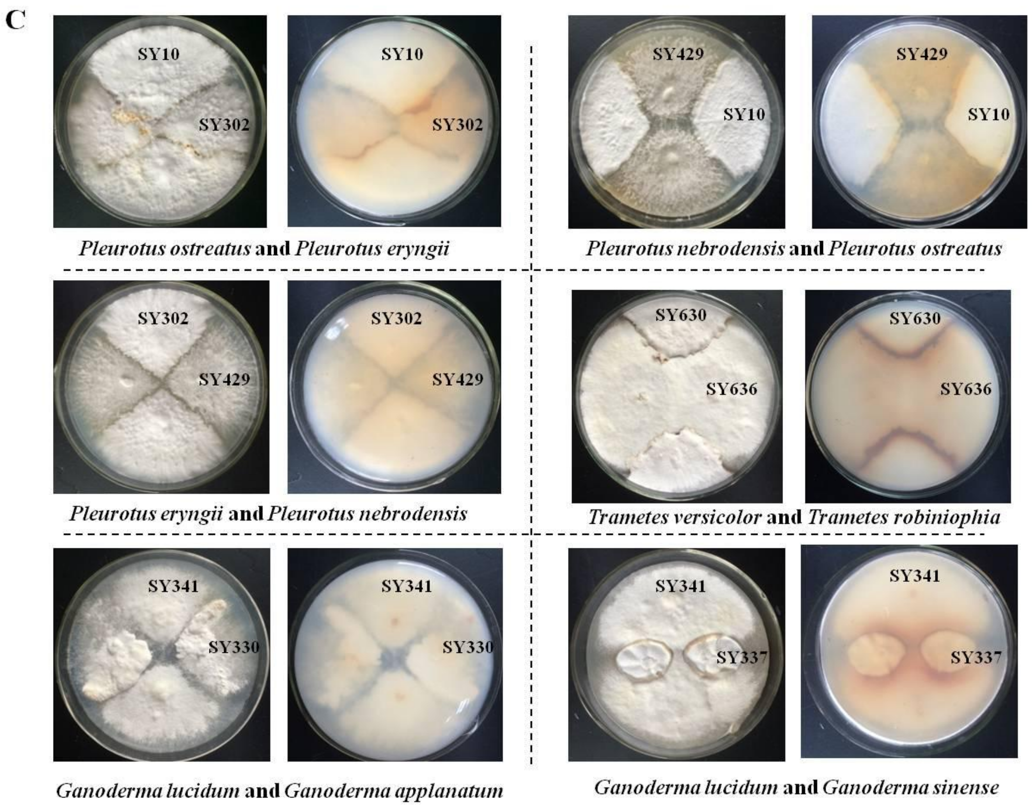

Figure S1. Colony morphologies of the representative co-cultures of basidiomycetes on agar plates. (A) Co-cultures (different order); (B) Co-cultures (different family); (C) Co-cultures (different species). For each pair, the left photo was taken from the top of plate and the right photo was taken from the bottom of plate.

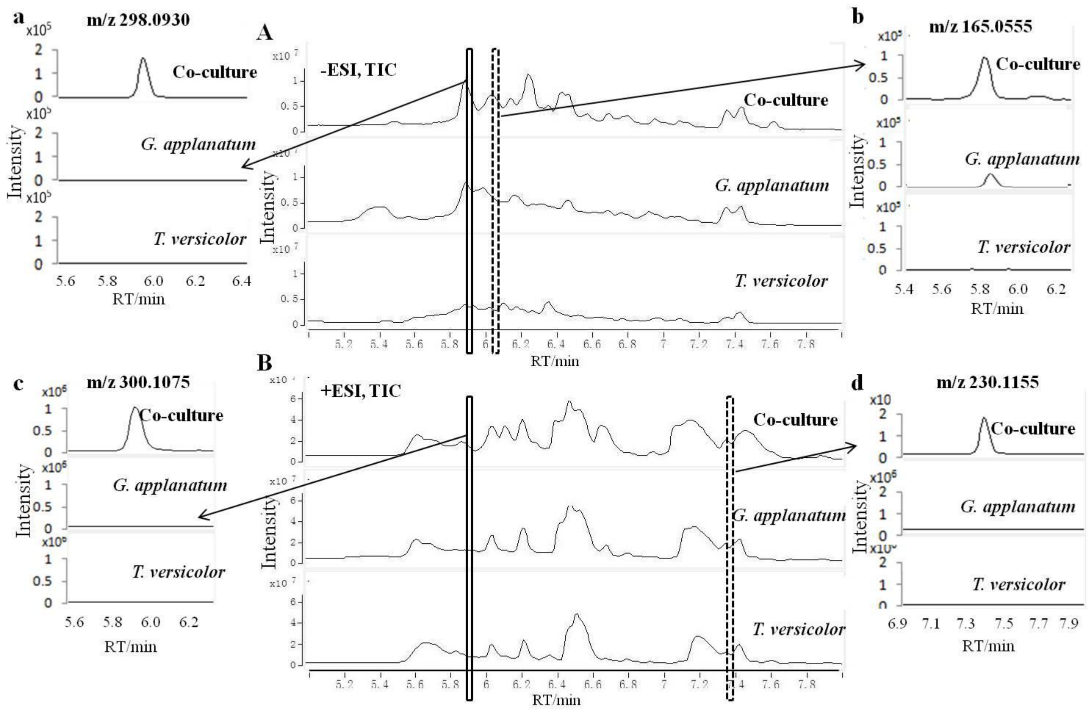

Figure S2. Comparison of total ion chromatograms (TICs) between co-culture of *T.versicolor* with *G. applanatum* and their corresponding mono-cultures on the day 23. (A) TICs of co-culture and mono-cultures were acquired by LC-MS in the negative mode. (a) and (b) was extracted ion chromatograms (EICs) of m/z 298.0930 and 165.0555; (B) TICs of co-culture and mono-cultures were acquired by LC-MS in the positive mode. (c) and (d) was EICs of m/z 300.1075 and 230.1155.

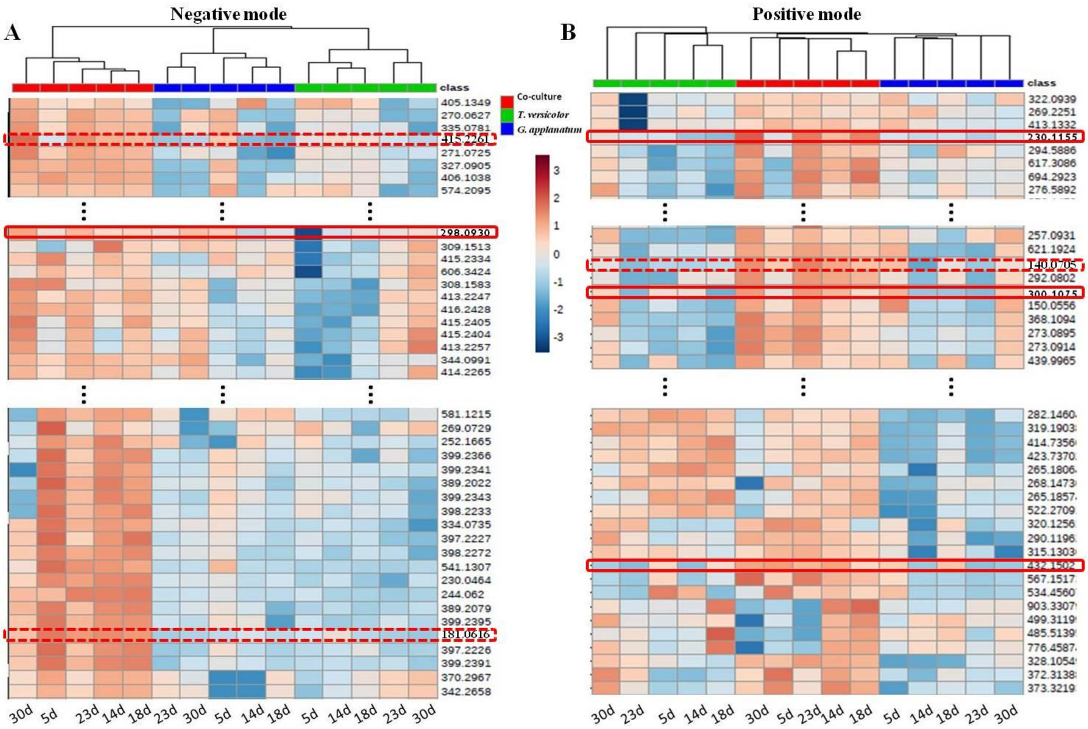

Figure S3. Heatmap analysis of co-culture of *T. versicolor* and *G. applanatum* and their corresponding mono-cultures at different time points (5, 14, 18, 23 and 30 days). (A) Heatmap of the features analyzed by LC-MS in the negative mode; (B) Heatmap of the features analyzed by LC-MS in the positive mode. The red squares were the representative features with significant difference between co-culture and mono-cultures, and dotted line squares were features only detected by heatmap analysis. Data was acquired from three independent biological replicates and two analytical replicates.

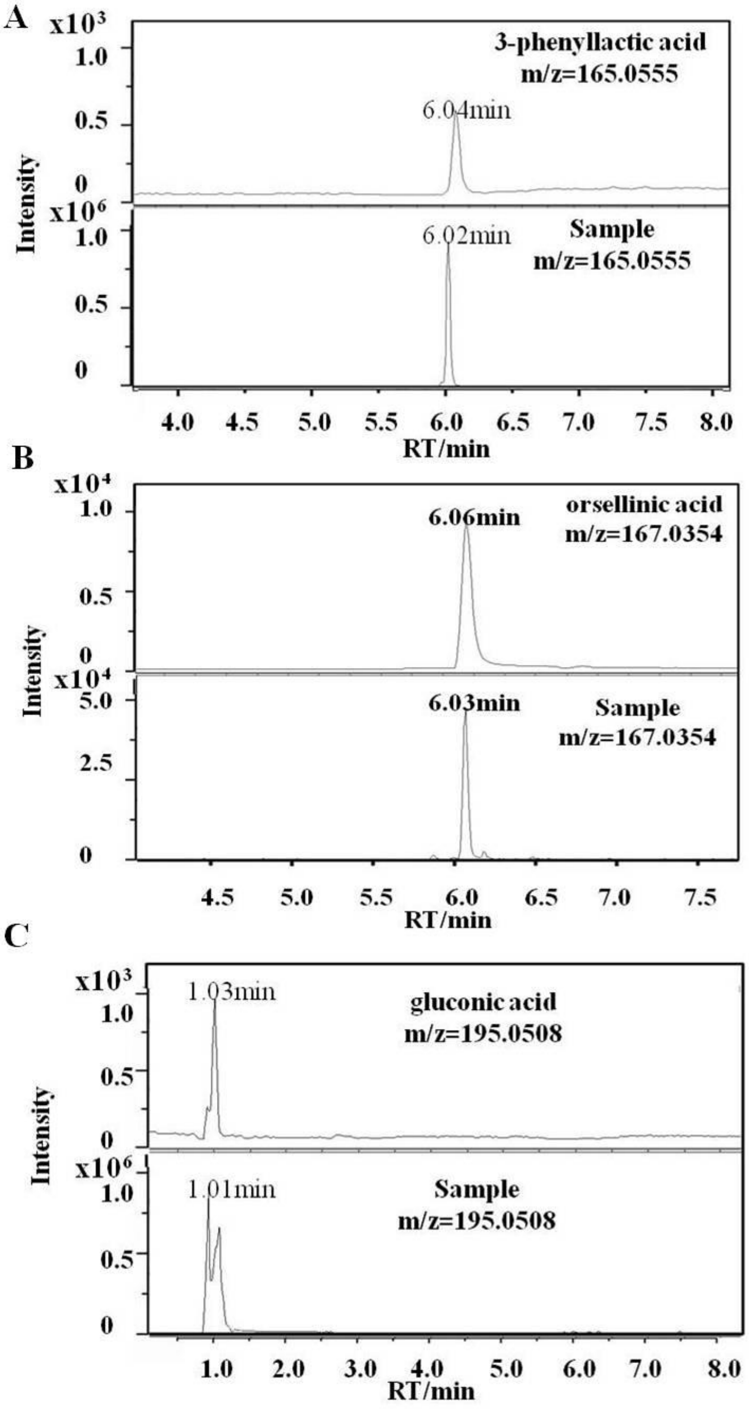

Figure S4. A comparison of retention time of three carboxylic acids between the experimental data and standards data. The extracted ion chromatograms was acquired by LC-MS in the negative mode. (A) 3-Phenyllactic acid; (B) orsellinic acid and (C) gluconic acid. Up panel was the standards and down panel was the compounds extracted from the co-culture of *T. versicolor* and *G. applanatum* on the day 9.

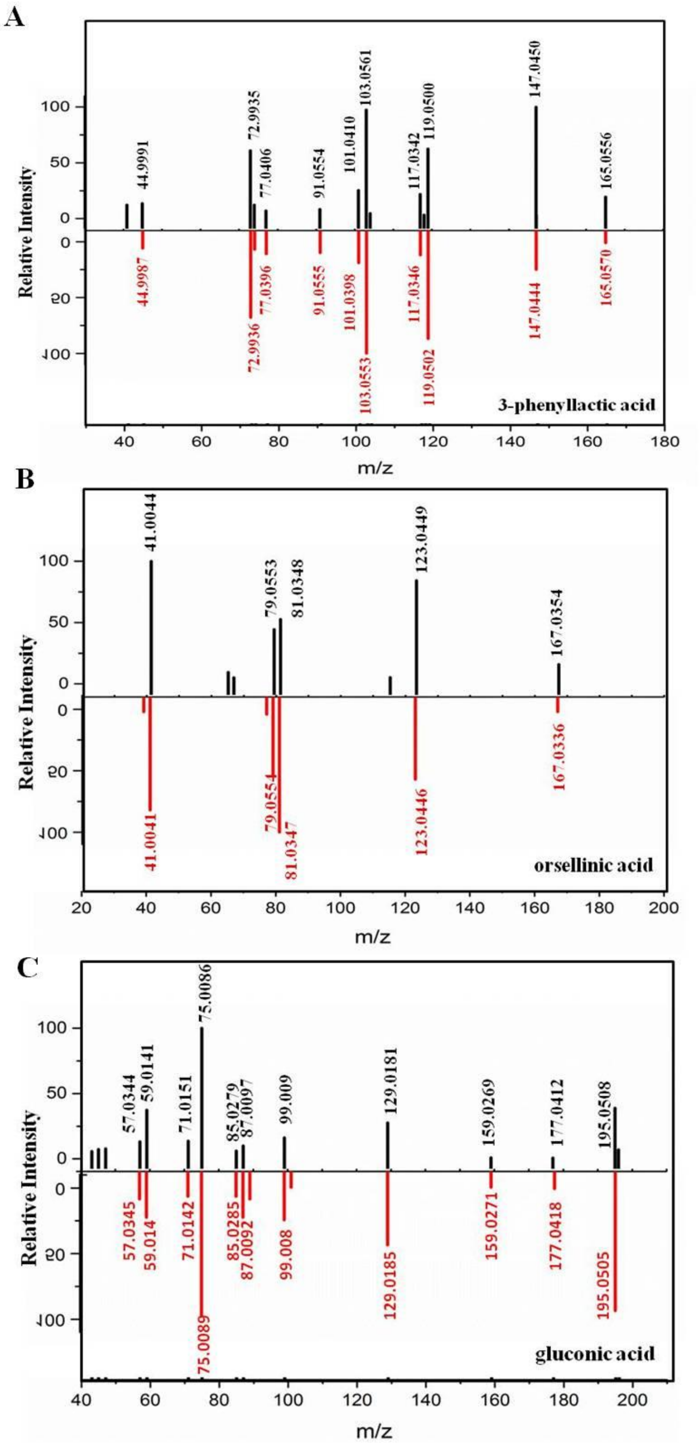

Figure S5. A comparison of MS/MS pattern of three carboxylic acids between the experimental data (black) and METLIN data (red). The mass spectral data was acquired by LC-MS/MS in the negative mode. (A) m/z 165.0555 matched with 3-phenyllactic acid; (B) m/z 167.0354 matched with orsellinic acid; (C) m/z 195.0508 matched with gluconic acid.

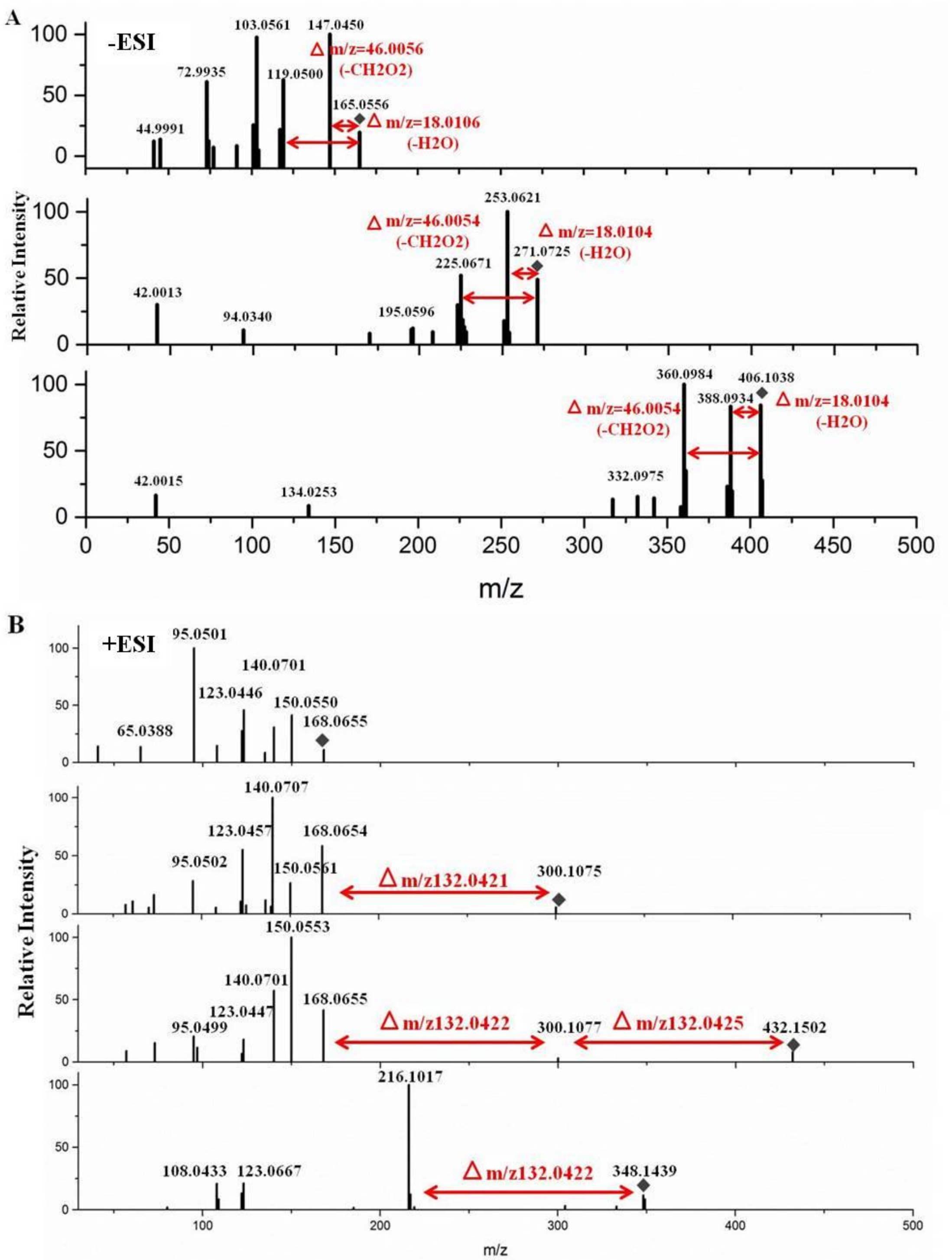

A
-ESI
Relative Intensity
m/z
B
+ESI

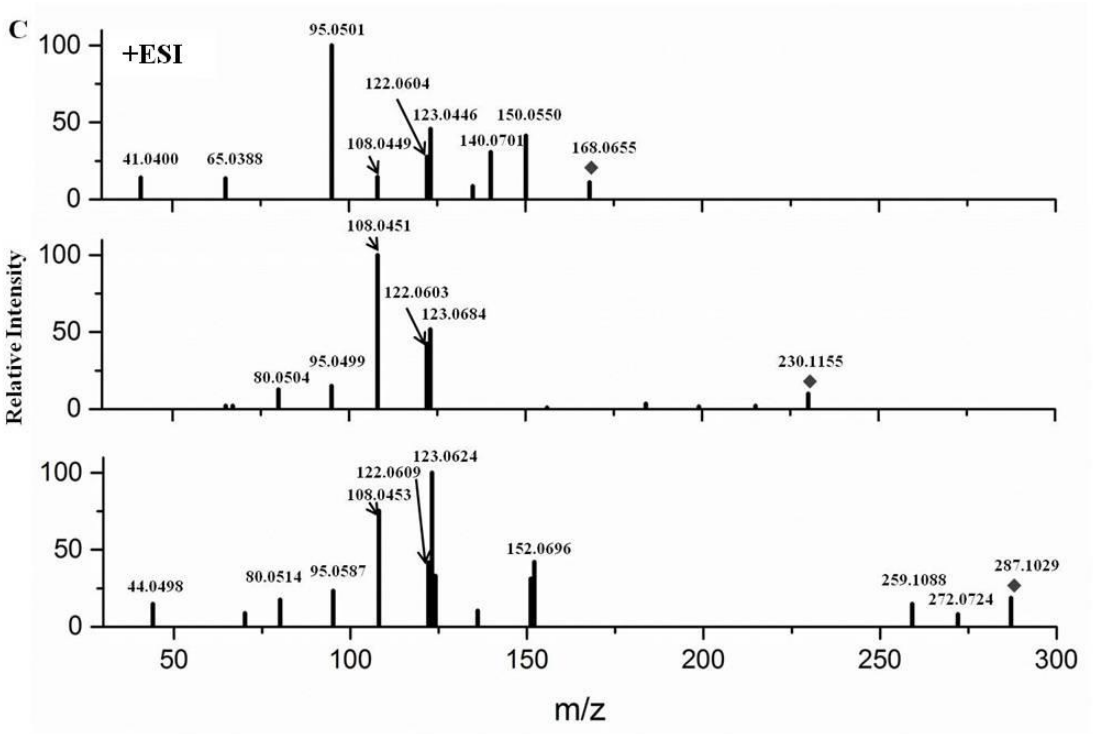

Figure S6. A comparison of MS/MS pattern of the features revealed by molecular network analysis. The mass spectral data was acquired by LC-MS/MS. (A) MS/MS spectra of m/z 165.0555, 271.0725, 406.1038 in the negative mode; (B) MS/MS spectra of xylosides (m/z 300.1075, 432.1502 and 348.1439) and m/z 168.0655 in the positive mode; (C) MS/MS spectra of m/z 168.0655 and its clusters (m/z 230.1155 and 287.1029) in the positive mode, and black arrow points out the same fragment ions generated from different parent ions.

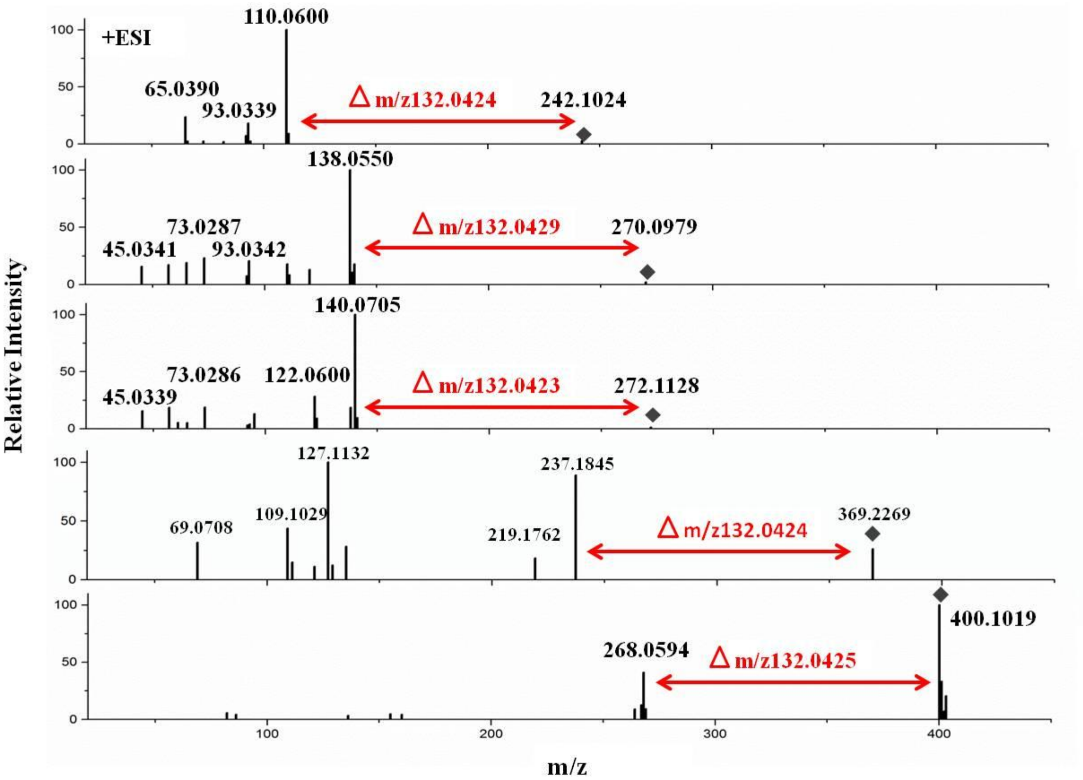

Figure S7. The xylosides were revealed by neutral loss scanning for a loss of 132 Dalton by LC-QQQ-MS. The figure shows that their MS/MS spectra were confirmed by high resolution LC-TOFMS in the positive mode.

^{1}H spectrum of compound **2**

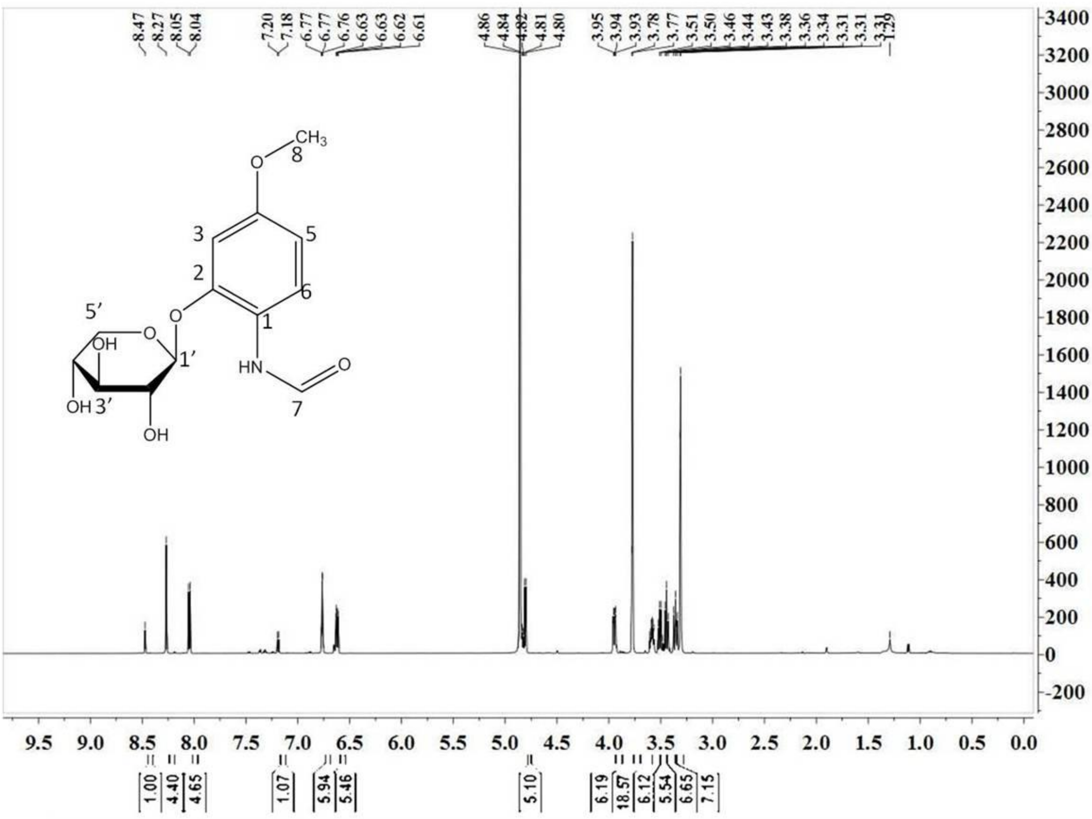

^{13}C spectrum of compound **2**

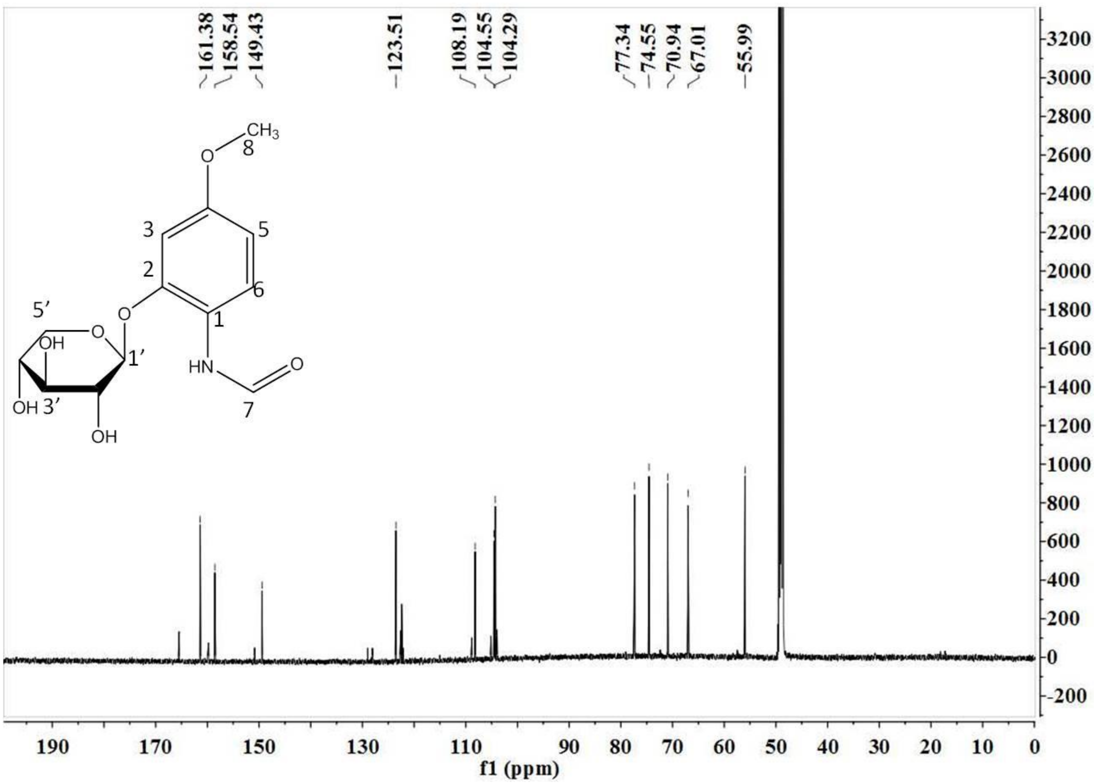

HMBC spectrum of compound **2**

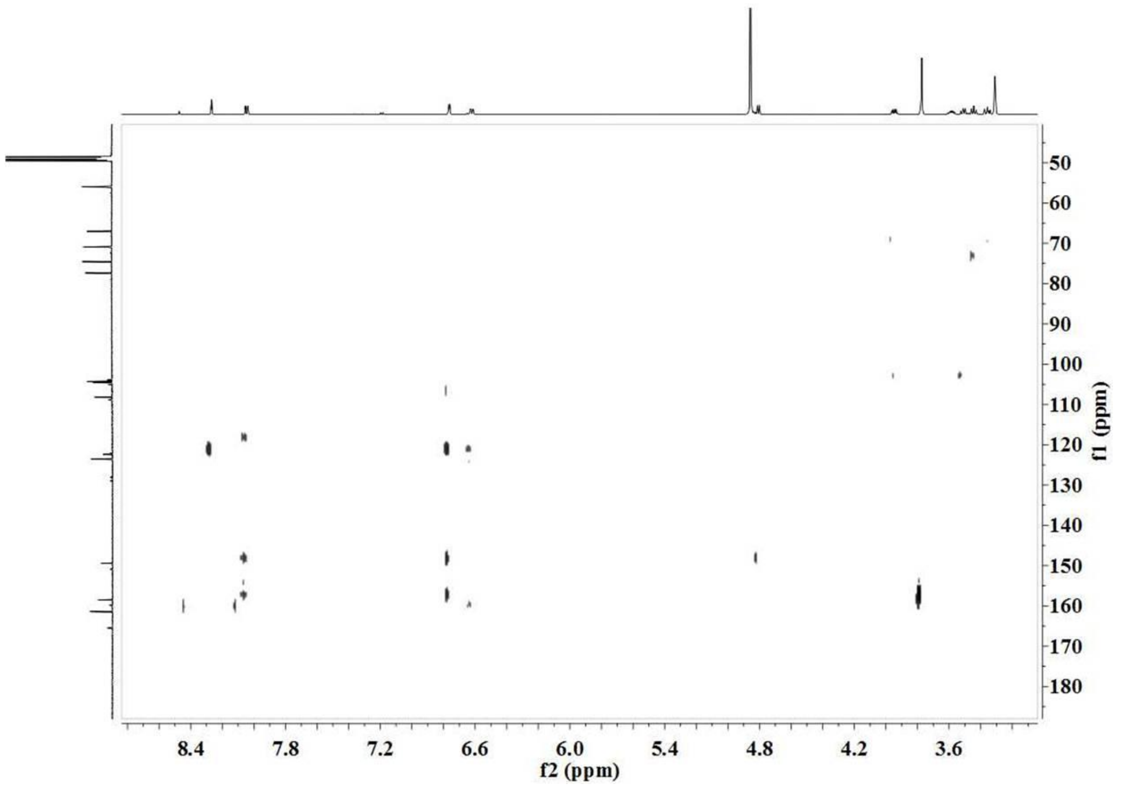

COSY spectrum of compound **2**

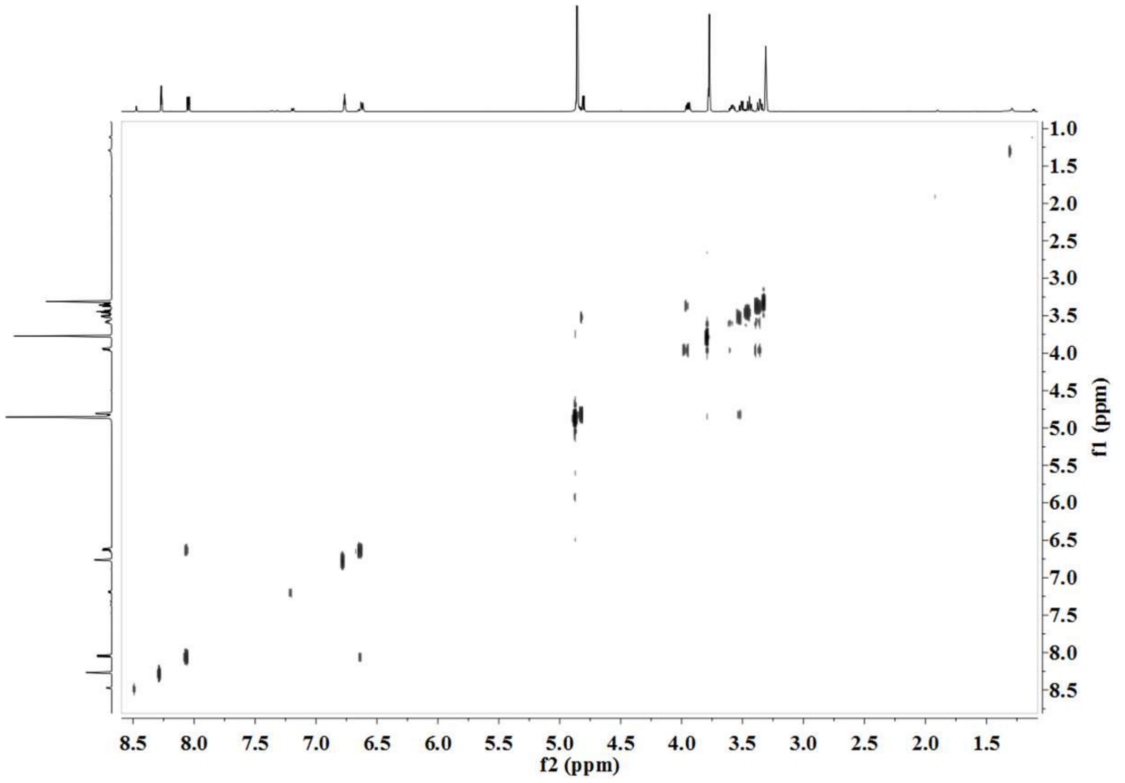

HSQC spectrum of compound **2**

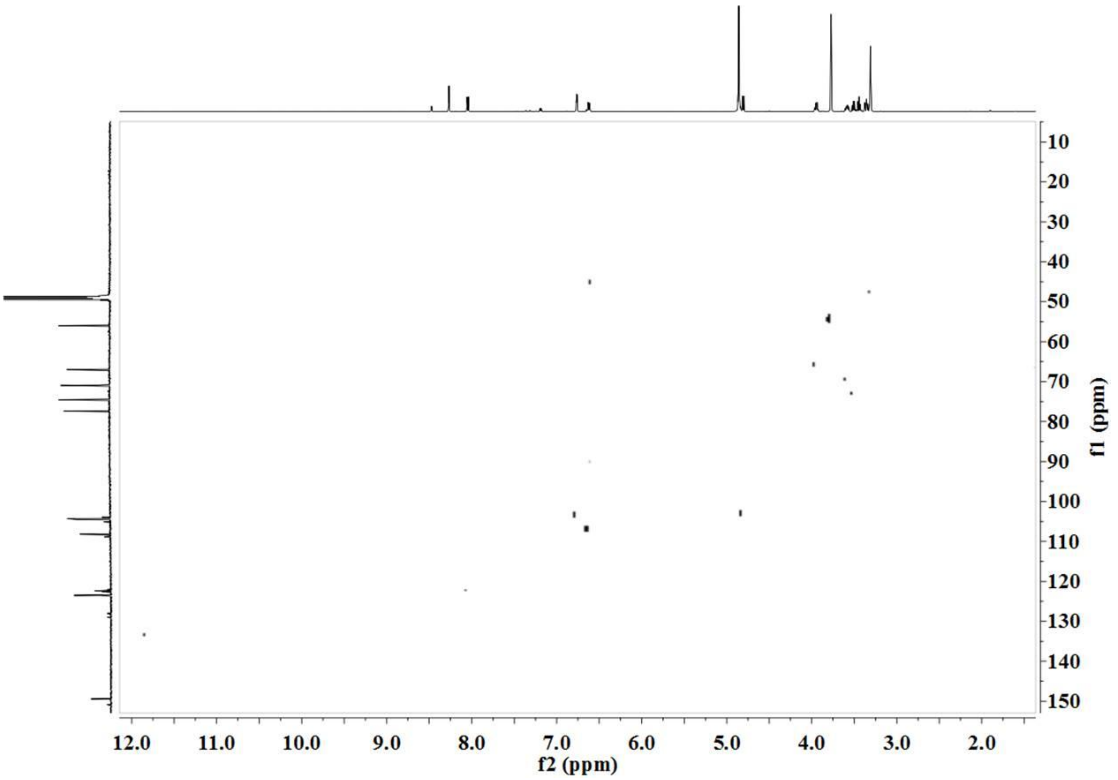

DEPT90° spectrum of compound **2**

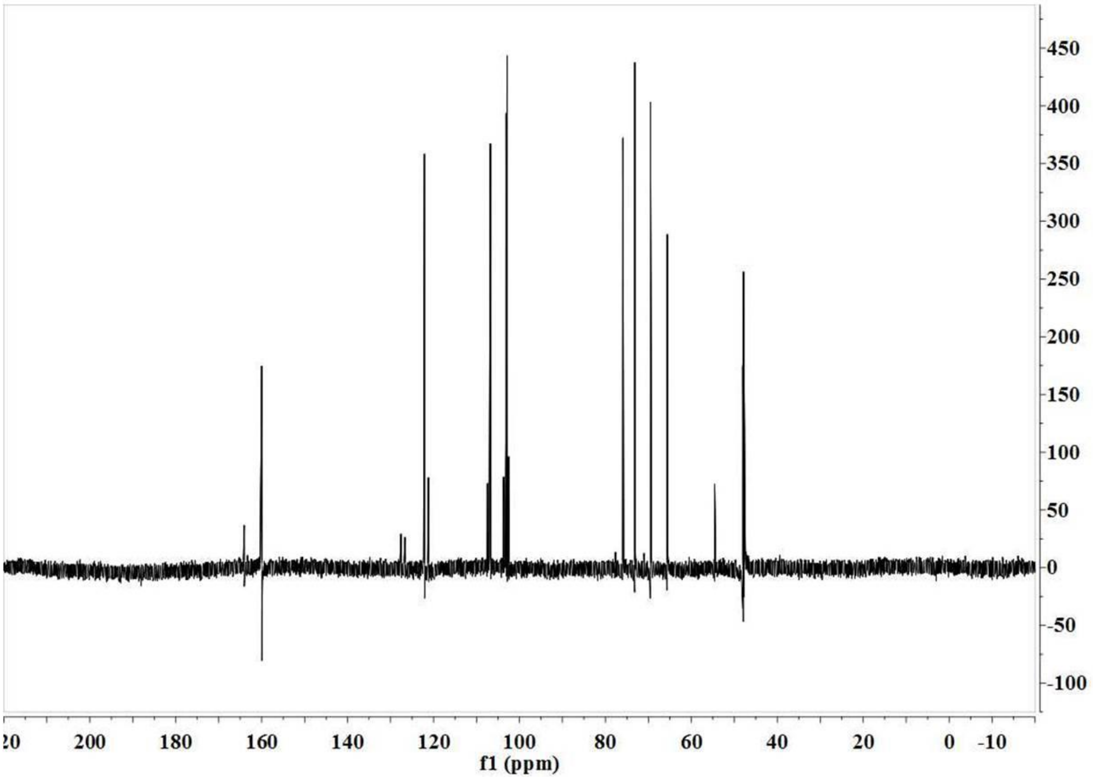

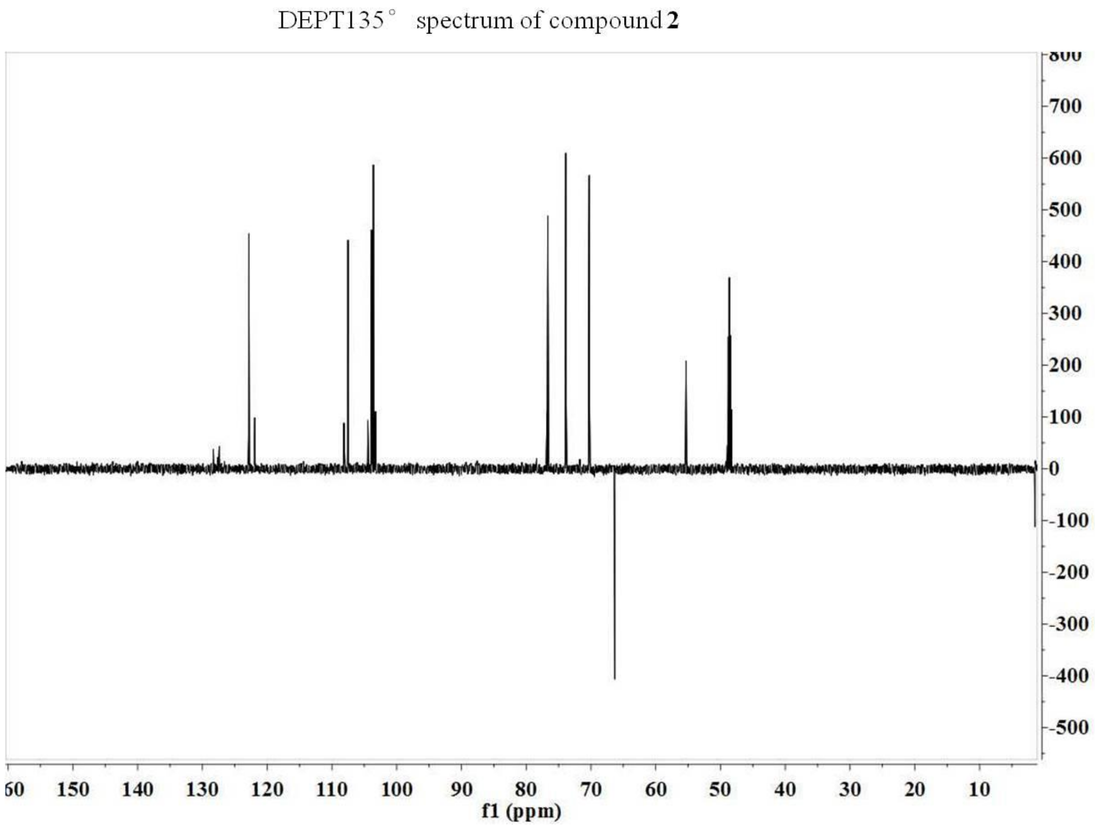

Figure S8. NMR spectra of compound **2.**

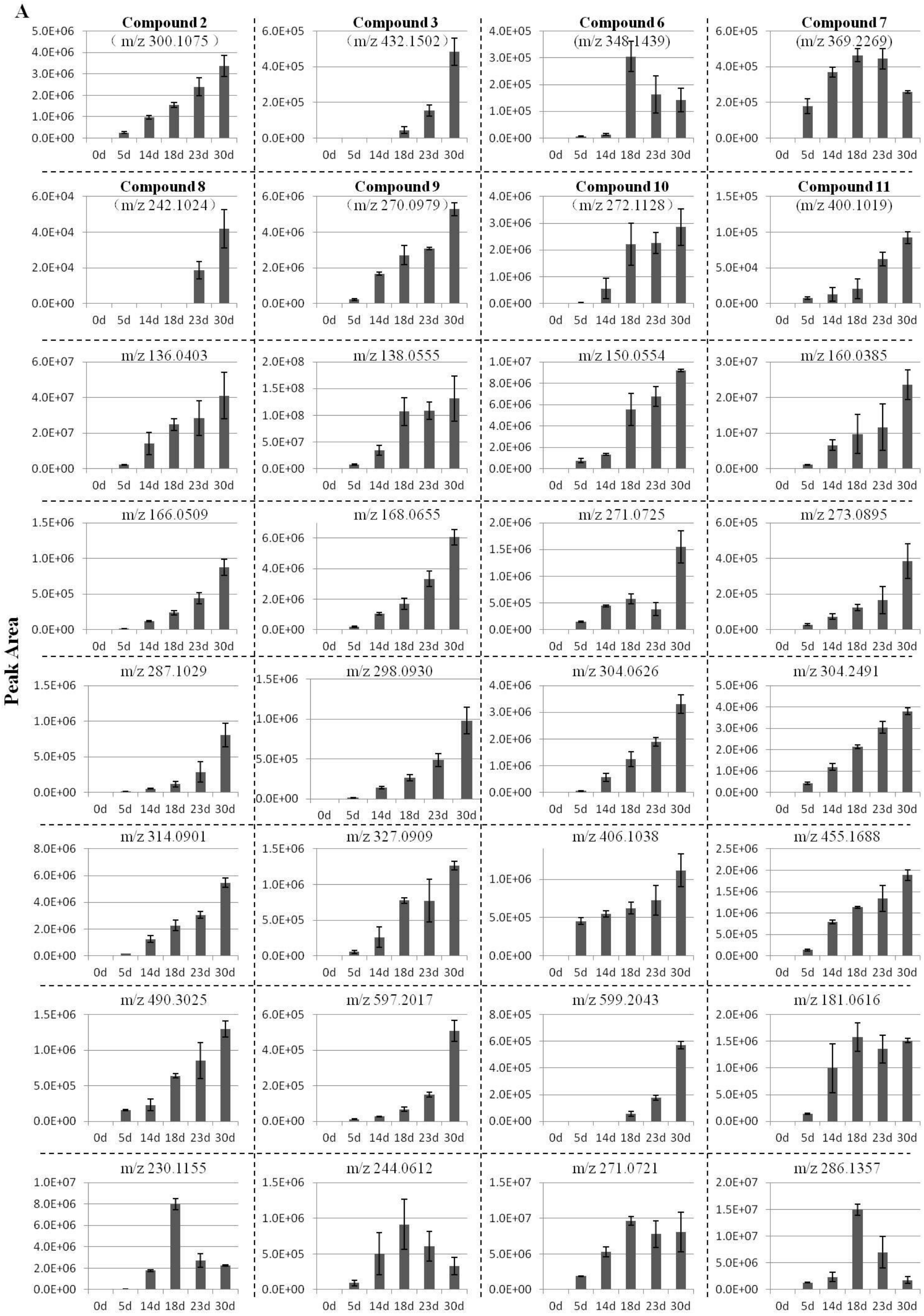
A
Peak Area
Compound 2
(m/z 300.1075)
Compound 3
(m/z 432.1502)
Compound 6
(m/z 348.1439)
Compound 7
(m/z 369.2269)
Compound 8
(m/z 242.1024)
Compound 9
(m/z 270.0979)
Compound 10
(m/z 272.1128)
Compound 11
(m/z 400.1019)
m/z 136.0403
m/z 138.0555
m/z 150.0554
m/z 160.0385
m/z 166.0509
m/z 168.0655
m/z 271.0725
m/z 273.0895
m/z 287.1029
m/z 298.0930
m/z 304.0626
m/z 304.2491
m/z 314.0901
m/z 327.0909
m/z 406.1038
m/z 455.1688
m/z 490.3025
m/z 597.2017
m/z 599.2043
m/z 181.0616
m/z 230.1155
m/z 244.0612
m/z 271.0721
m/z 286.1357
0d
5d
14d
18d
23d
30d

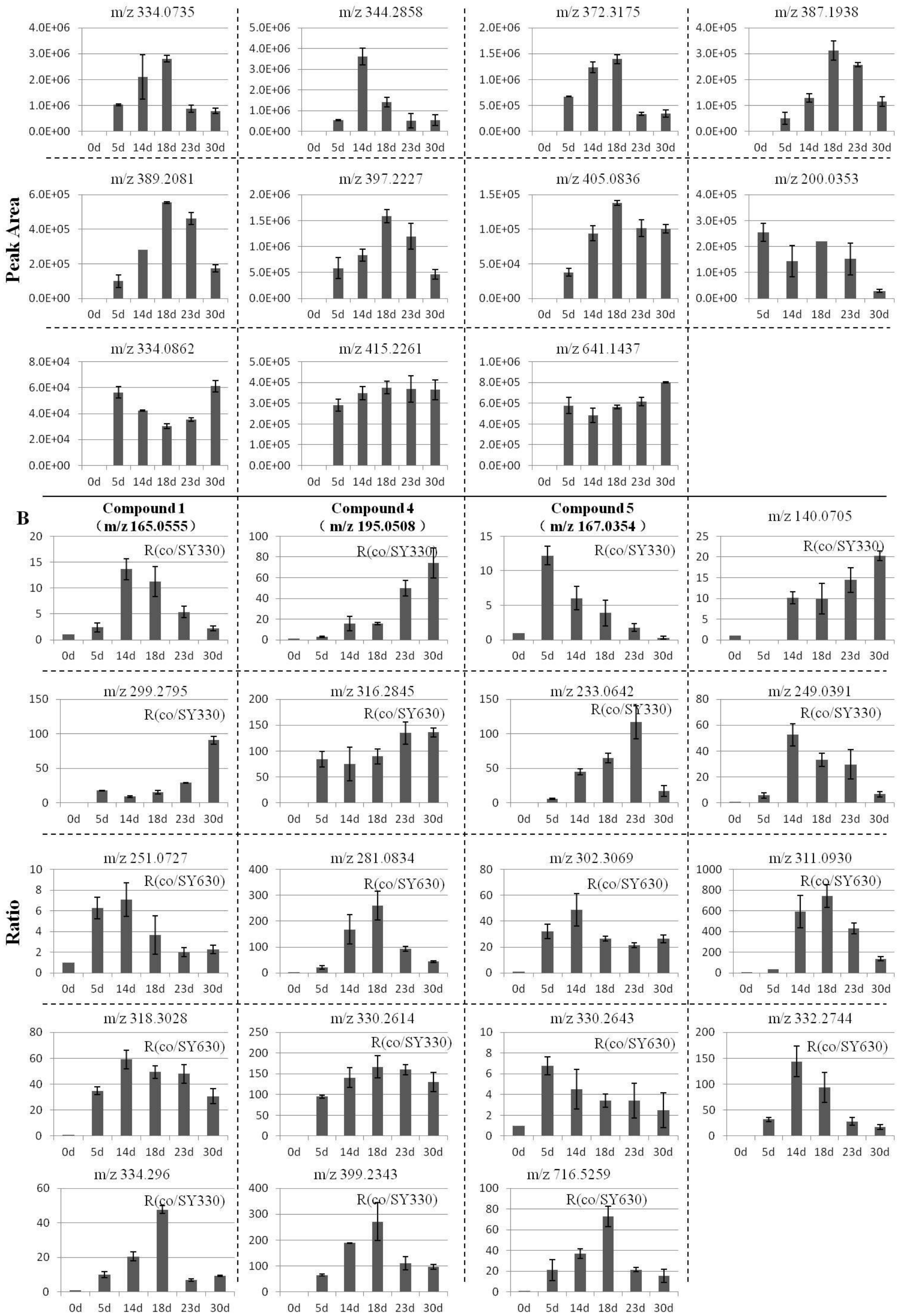

Figure S9. Dynamics of induced features accumulation in fungal co-culture from the day 0 to day 30. (A) Induced newly features; (B) Increased features in the co-culture. R(co/SY330) and R(co/SY630) means the ratio of the abundance in the co-culture to the mono-culture of *G. applanatum* SY330 and the ratio of the abundance in the co-

culture to the mono-culture of *T. versicolor* SY630, respectively. Data show the mean with error bars indicating standard deviation calculated from three independent biological replicates.

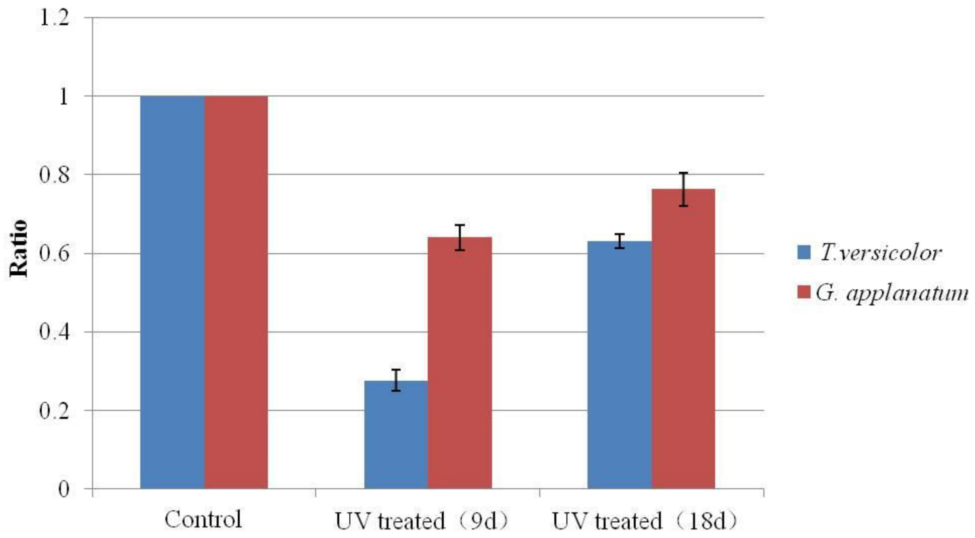

Figure S10. Comparison of mycelium wet weight between UV-treated fungi and the control on the days 9 and 18. The y axis is the ratio of the wet weight of UV-treated *G.applanatum* or *T. versicolor* to the wet weight of fungi without treatment (the control). The average value for the control was set to 1. Data show the mean with error bars indicating standard deviation calculated from three independent biological replicates.

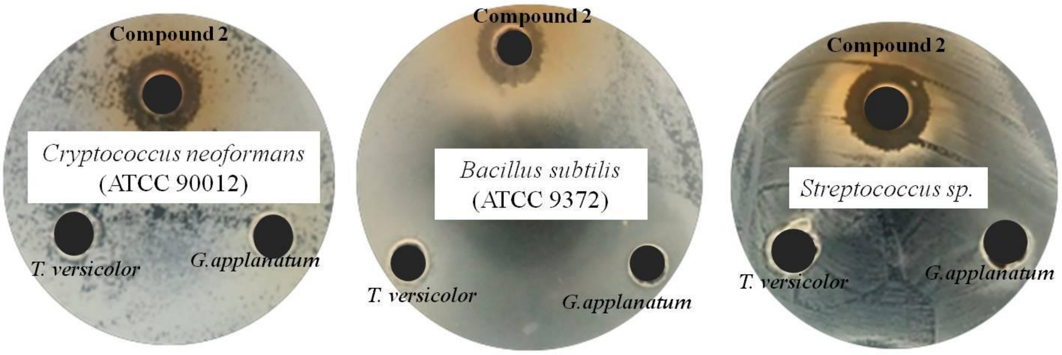

Figure S11. Antimicrobial activities of compound **2** by disc diffusion assay. *Cryptococcus neoformans*, *Bacillus subtilis* and *Streptococcus* sp. were employed as antagonistic pathogenic microbe. 170 μM of compound **2** was added to sterile borer, and 20-fold concentrated supernatant from mono-cultures of *T. versicolor* and *G.applanatum* were used as the control. The incubation was 12 hours, and zone of inhibition of compound 2 was about 1.0 cm and the control was zero.

Table S1. A list of seventeen basidiomycetes in this research. They were either deposited at the Shandong Province Key Lab of Applied Mycology or stored in China General Microbiological Culture Collection Center (CGMCC).

Strains	Strain ID	Order	Family	Genus	Wood-decaying
Pleurotus ostreatus	SY10	Agaricales	Pleurotaceae	Pleurotus	Yes
Lentinus edodes	SY103	Agaricales	Marasmiaceae	Lentinula	Yes
Agaricus bisporus	SY153	Agaricales	Agaricaceae	Agaricus	No
Flammulina velutipes	SY204	Agaricales	Physalacriaceae	Flammulina	Yes
Grifola frondosa	SY251	Polyporales	Meripilaceae	Grifola	Yes
Pleurotus eryngii	SY302	Agaricales	Pleurotaceae	Pleurotus	Yes
Ganoderma applanatum	SY330	Polyporales	Ganodermataceae	Ganoderma	Yes
Ganoderma sinense	SY337	Polyporales	Ganodermataceae	Ganoderma	Yes
Ganoderma lucidum	SY341	Polyporales	Ganodermataceae	Ganoderma	Yes
Pleurotus nebrodensis	SY429	Agaricales	Pleurotaceae	Pleurotus	Yes
Phellinus igniarius	SY489	Hymenochaetales	Hymenochaetaceae	Phellinus	Yes
Cordyceps militaris	SY501	Hypocreales	Clavicipitaceae	Cordyceps	No
Boletus edulis	SY613	Boletales	Boletaceae	Boletus	Yes
Trametes versicolor	SY630	Polyporales	Polyporaceae	Trametes	Yes
Trametes robiniophia	SY636	Polyporales	Polyporaceae	Trametes	Yes
Schizophyllum commune	SY658	Agaricales	Schizophyllaceae	Schizophyllum	Yes
Sclerotium xylariae nigripis	SY687	Xylariales	Xylariaceae	Sclerotium	Yes

Table S2. List of induced features in the co-culture of *T. versicolor* and *G.applanatum.*

m/z (-)	RT/min	Difference	m/z (-)	RT/min	Difference	m/z (+)	RT/min	Difference
195.0508	1.09	increased	334.0735	6.34	newly synthesized	300.1075	5.86	newly synthesized
181.0616	2.13	newly synthesized	327.0909	6.35	newly synthesized	432.1502	5.87	newly synthesized
304.0626	2.13	newly synthesized	405.0836	6.3	newly synthesized	599.2043	5.88	newly synthesized
314.0901	2.24	newly synthesized	490.3025	6.37	newly synthesized	150.0554	5.99	newly synthesized
136.0403	2.57	newly synthesized	251.0727	6.68	increased	304.2491	6.01	newly synthesized
271.0721	5.48	newly synthesized	399.2343	7.61	increased	287.1029	6.05	newly synthesized
271.0725	5.71	newly synthesized	397.2227	7.61	newly synthesized	330.2643	6.08	increased
244.0612	5.36	newly synthesized	387.1938	7.61	newly synthesized	334.296	6.09	increased
406.1038	5.79	newly synthesized	389.2081	7.62	newly synthesized	332.2744	6.15	increased
166.0509	5.86	newly synthesized	**m/z (+)**	**RT**	**Difference**	318.3028	6.15	increased
298.093	5.87	newly synthesized	249.0391	1.1	increased	316.2845	6.17	increased
597.2017	5.88	newly synthesized	233.0642	1.18	increased	348.1439	6.22	newly synthesized
455.1688	5.86	newly synthesized	242.1024	1.42	newly synthesized	369.2269	6.24	newly synthesized
165.0555	6.02	increased	272.1128	2.09	newly synthesized	334.0862	6.26	newly synthesized
167.0354	6.05	increased	140.0705	2.19	increased	400.1019	6.35	newly synthesized
330.2614	6.13	increased	270.0979	2.27	newly synthesized	299.2795	6.43	Increased
311.0930	6.17	increased	160.0385	2.59	newly synthesized	344.2858	7.12	newly synthesized
200.0353	6.17	newly synthesized	138.0555	2.7	newly synthesized	230.1155	7.46	newly synthesized
281.0834	6.2	increased	286.1357	5.03	newly synthesized	716.5259	7.54	Increased
641.1437	6.26	newly synthesized	273.0895	5.68	newly synthesized	302.3069	7.54	Increased
415.2261	6.27	newly synthesized	168.0655	5.87	newly synthesized	372.3175	8.16	newly synthesized

Table S3. ^{1}H-NMR and ^{13}C-NMR data for compound **2**, δ in ppm

	δ_H	δ_C
1		122.4
2		149.0
3	6.76 (1H, d)	104.5
4		159.0
5	6.62 (1H, q)	108.4
6	8.04 (1H, d)	123.9
7	8.27 (1H, brs)	161.4
8	3.77 (3H, brs)	56.9
1'	4.81 (1H, d)	104.4
2'	3.50 (1H, d)	74.9
3'	3.44 (1H, s)	77.5
4'	3.58 (1H, m)	71.3
5'	3.36 (1H, s)	67.4
	3.95 (1H, m)	

Table S4. Bioinformatics analysis of the potential xylosyltransferases encoded by *T.versicolor*. Only the potential query protein with an identity of ≥20% to the database and the query length no less than 120 bps are shown.

No	Locus tag	Protein annotation	length	NCBI Accession	query length	Identity (%)	Query start	Query stop	objective sequences ID	objective sequence function
1	TRAVE DRAFT_ 31538	capsular associated protein	630bp	GI|636624487|ref|XP_008043378.1|	645	27.29	6	612	GI|927760026|gb|KPA43099.1|	beta- -xylosyltransferase 1 [Fusarium langsethiae]
					641	28.71	30	628	GI|647399448|emb|CDR44236.1|	RHTO0S09e01508g1_1 [*Rhodotorula toruloides*]
					604	33.61	65	623	GI|647397492|emb|CDR40532.1|	RHTO0S05e04610g1_1 [*Rhodotorula toruloides*]
					600	26.17	59	626	GI|751352302|gb|KIL90024.1|	beta- -xylosyltransferase 1 [*Fusarium avenaceum*]
					600	23.17	65	626	GI|949392729|dbj|GAP84327.1|	putative glycosyltransferase family 90 protein [*Rosellinia necatrix*]
					599	50.58	60	626	GI|924113091|emb|CUA78292.1|	Beta-1,2-xylosyltransferase 1 [*Rhizoctonia solani*]
					599	28.05	60	628	GI|816192268|emb|CRG85785.1|	Beta-1,2-xylosyltransferase 1 [*Talaromyces islandicus*]
					598	27.26	60	626	GI|358386117|gb|EHK23713.1|	glycosyltransferase family 90 protein [*Trichoderma virens Gv29-8*]
					598	27.26	60	626	GI|927424180|ref|XP_013957921.1|	glycosyltransferase family 90 protein [*Trichoderma virens Gv29-8*]
					598	26.76	64	626	GI|927760719|gb|KPA43756.1|	xylosyltransferase 1 [*Fusarium langsethiae*]
					597	28.81	58	626	GI|358398656|gb|EHK48007.1|	glycosyltransferase family 90 protein [*Trichoderma atroviride* IMI 206040]
					597	28.81	58	626	GI|927400707|ref|XP_013946199.1|	glycosyltransferase family 90 protein [*Trichoderma atroviride* IMI 206040]
					592	29.39	65	625	GI|647397714|emb|CDR40950.1|	RHTO0S05e09538g1_1 [*Rhodotorula toruloides*]
					590	27.8	71	626	GI|358377868|gb|EHK15551.1|	glycosyltransferase family 90 protein [*Trichoderma virens Gv29-8*]
					590	27.8	71	626	GI|927406821|ref|XP_013949749.1|	glycosyltransferase family 90 protein [*Trichoderma virens Gv29-8*]
					589	49.41	57	615	GI|751836596|emb|CEL53282.1|	Beta-1,2-xylosyltransferase 1 OS=Cryptococcus neoformans var, neoformans serotype D (strain JEC21 / ATCC MYA-565) GN=CXT1 PE=1 SV=1 [*Rhizoctonia solani* AG-1 IB]
					586	27.13	71	628	GI|358386962|gb|EHK24557.1|	glycosyltransferase family 90 protein [*Trichoderma virens* Gv29-8]
					586	27.13	71	628	GI|927425858|ref|XP_013958760.1|	glycosyltransferase family 90 protein [*Trichoderma virens* Gv29-8]
					585	30.09	65	626	GI|647402717|emb|CDR48932.1|	Weakly similar to uniprot|Q5K8R6 Cryptococcus
					583	29.67	63	629	GI|647397729|emb|CDR40965.1|	RHTO0S05e09890g1_1 [*Rhodotorula toruloides*]
					582	28.35	65	629	GI|74682361|sp|Q5K8R6.1|CXT1_CRYNJ	RecName: Full=Beta-1,2-xylosyltransferase 1; Short=Cxt1p
					582	28.35	65	629	GI|58261216|ref|XP_568018.1|	cryptococcal xylosyltransferase 1 [*Cryptococcus neoformans* var. neoformans JEC21]
					582	28.35	65	629	GI|57230100|gb|AAW46501.1|	cryptococcal xylosyltransferase 1 [*Cryptococcus neoformans* var.

										neoformans JEC21]
					582	28.35	65	629	GI|146742896|gb|ABQ42713.1|	beta-1,2 xylosyltransferase 1 [*Cryptococcus neoformans* var. neoformans]
					578	27.85	60	622	GI|647397408|emb|CDR40393.1|	RHTO0S05e02784g1_1 [*Rhodotorula toruloides*]
					577	29.81	65	630	GI|647397582|emb|CDR40723.1|	RHTO0S05e06634g1_1 [*Rhodotorula toruloides*]
					577	26.69	54	623	GI|949389023|dbj|GAP88019.1|	putative glycosyltransferase family 90 protein [*Rosellinia necatrix*]
					576	27.08	65	628	GI|924115849|emb|CUA75853.1|	hypothetical protein RSOLAG22IIIB_01853 [*Rhizoctonia solani*]
					574	28.57	65	627	GI|647397677|emb|CDR40913.1|	RHTO0S05e08724g1_1 [*Rhodotorula toruloides*]
					574	27.18	65	628	GI|751840883|emb|CEL55146.1|	hypothetical protein RSOLAG1IB_01154 [*Rhizoctonia solani AG-1 IB*]
					572	28.67	63	625	GI|358386481|gb|EHK24077.1|	glycosyltransferase family 90 protein [Trichoderma virens Gv29-8]
					572	28.67	63	625	GI|927424890|ref|XP_013958276.1|	glycosyltransferase family 90 protein [*Trichoderma virens Gv29-8*]
					546	26.74	82	622	GI|927751359|gb|KPA36041.1|	beta- -xylosyltransferase 1 [*Fusarium langsethiae*]
					506	24.31	62	560	GI|927750330|gb|KPA35555.1|	beta- -xylosyltransferase 1 [*Fusarium langsethiae*]
2	TRAVE DRAFT_ 31521	hypothetical protein	558bp	GI|636624477|ref|XP_008043373.1|	556	49.82	6	558	GI|751836596|emb|CEL53282.1|	Beta-1,2-xylosyltransferase 1 [*Rhizoctonia solani* AG-1 IB]
3	TRAVE DRAFT_ 56171	UDP-Glycosyltransferase/glycogen phosphorylase	515bp	GI|636605689|ref|XP_008033979.1|	331	25.98	193	509	GI|5802783|gb|AAD51778.1|AF116858_1	zeatin O-xylosyltransferase [*Phaseolus vulgaris*]
					331	25.98	193	509	GI|6226511|sp|P56725.1|ZOX_PHAVU	Full=Zeatin O-xylosyltransferase; AltName: Full=Zeatin O-beta-D-xylosyltransferase [*Phaseolus vulgaris*]
					293	27.3	239	503	GI|15234056|ref|NP_192016.1|	UDP-glycosyltransferase 72B1 [*Arabidopsis thaliana*]
					293	27.3	239	503	GI|332656577|gb|AEE81977.1|	UDP-glycosyltransferase 72B1 [*Arabidopsis thaliana*]
					229	25.33	263	477	GI|75311632|sp|Q9LVW3.1|AXYLT_ARATH	RecName: Full=Anthocyanidin 3-O-glucoside 2'''-O-xylosyltransferase; Short=A3G2"XylT [*Arabidopsis thaliana*]
4	TRAVE DRAFT_ 73641	hypothetical protein	444bp	GI|636620747|ref|XP_008041508.1|	317	25.87	130	414	GI|829746565|ref|XP_012610675.1|	PREDICTED: xylosyltransferase 1 [*Microcebus murinus*]
					286	23.78	90	332	GI|512987849|ref|XP_004855851.1|	PREDICTED: xylosyltransferase 1 [*Heterocephalus glaber*]
					267	22.47	90	306	GI|694962633|ref|XP_009428658.1|	PREDICTED: xylosyltransferase 1 isoform X1 [*Pan troglodytes*]
					267	23.22	90	306	GI|795246872|ref|XP_011917739.1|	PREDICTED: xylosyltransferase 1 [*Cercocebus atys*]
					267	23.22	90	306	GI|967501808|ref|XP_014981193.1|	PREDICTED: xylosyltransferase 1 [*Macaca mulatta*]
					266	21.05	90	306	GI|837808540|ref|XP_012782911.1|	PREDICTED: xylosyltransferase 1 [*Ochotona princeps*]
					266	23.68	90	306	GI|71164803|sp|Q86Y38.1|XYLT1_HUMAN	RecName: Full=Xylosyltransferase 1; AltName: Full=Peptide O-xylosyltransferase 1; AltName: Full=Xylosyltransferase I; Short=XT-I; Short=XylT-I [*Homo sapiens*]
					266	23.68	90	306	GI|162317952|gb|AAI56040.1|	Xylosyltransferase I, partial [*synthetic construct*]
					266	23.68	90	306	GI|28172878|emb|CAD62248.1|	xylosyltransferase I [*Homo sapiens*]

					266	23.68	90	306	GI|767987432|ref|XP_011520876.1|	PREDICTED: xylosyltransferase 1 isoform X1 [*Homo sapiens*]
					266	23.68	90	306	GI|28269693|ref|NP_071449.1|	xylosyltransferase 1 precursor [*Homo sapiens*]
					265	23.77	88	306	GI|795501572|ref|XP_011710287.1|	PREDICTED: LOW QUALITY PROTEIN: xylosyltransferase 1 [*Macaca nemestrina*]
					234	25.21	189	414	GI|71164803|sp|Q86Y38.1|XYLT1_HUMAN	RecName: Full=Xylosyltransferase 1; AltName: Full=Peptide O-xylosyltransferase 1; AltName: Full=Xylosyltransferase I; Short=XT-I; Short=XylT-I [*Homo sapiens*]
					234	25.21	189	414	GI|162317952|gb|AAI56040.1|	Xylosyltransferase I, partial [synthetic construct]
					234	25.21	189	414	GI|28172878|emb|CAD62248.1|	xylosyltransferase I [*Homo sapiens*]
					234	25.21	189	414	GI|767987432|ref|XP_011520876.1|	PREDICTED: xylosyltransferase 1 isoform X1 [*Homo sapiens*]
					234	25.21	189	414	GI|28269693|ref|NP_071449.1|	xylosyltransferase 1 precursor [*Homo sapiens*]
					219	21.46	136	306	GI|507701081|ref|XP_004644560.1|	PREDICTED: xylosyltransferase 1 [*Octodon degus*]
					215	23.72	189	390	GI|512987849|ref|XP_004855851.1|	PREDICTED: xylosyltransferase 1 [*Heterocephalus glaber*]
5	TRAVE DRAFT_ 62781	hypothetical protein	539bp	GI|636606177|ref|XP_008034223.1|	287	26.48	77	332	GI|829746565|ref|XP_012610675.1|	PREDICTED: xylosyltransferase 1 [*Microcebus murinus*]
					209	24.88	170	361	GI|296473377|tpg|DAA15492.1|	TPA: xylosyltransferase I [*Bos taurus*]
					209	24.88	170	361	GI|297490134|ref|XP_002698083.1|	PREDICTED: xylosyltransferase 1 [*Bos taurus*]
					209	24.88	170	361	GI|983007045|ref|XP_002703127.3|	PREDICTED: xylosyltransferase 1 [*Bos taurus*]
					206	25.24	170	361	GI|803250859|ref|XP_011985388.1|	PREDICTED: xylosyltransferase 1 isoform X2 [*Ovis aries musimon*]
6	TRAVE DRAFT_ 59053	hypothetical protein	420bp	GI|636616829|ref|XP_008039549.1|	272	26.1	143	391	GI|521027181|gb|EPQ08969.1|	Xylosyltransferase 2 [*Myotis brandtii*]
					239	24.69	171	391	GI|940773949|ref|XP_014302937.1|	PREDICTED: xylosyltransferase 2 isoform X1 [*Myotis lucifugus*]
					236	25.85	171	391	GI|554547365|ref|XP_005868235.1|	PREDICTED: xylosyltransferase 2 isoform X2 [*Myotis brandtii*]
					236	25.85	171	391	GI|946767238|ref|XP_014396660.1|	PREDICTED: xylosyltransferase 2 isoform X1 [*Myotis brandtii*]
7	TRAVE DRAFT_ 63062	hypothetical protein	551bp	GI|636608203|ref|XP_008035236.1|	487	30.8	16	488	GI|949393989|dbj|GAP83249.1|	putative wsc domain protein [*Rosellinia necatrix*]
					193	25.91	368	545	GI|908390298|gb|KND90936.1|	Xylosyltransferase oxt [*Tolypocladium ophioglossoides* CBS 100239]
					149	25.5	384	510	GI|972140057|gb|KUI66306.1|	Xylosyltransferase oxt [*Valsa mali*]
					130	27.69	383	506	GI|242008940|ref|XP_002425252.1|	Xylosyltransferase oxt, putative [*Pediculus humanus corporis*]
					130	27.69	383	506	GI|212509000|gb|EEB12514.1|	Xylosyltransferase oxt, putative [*Pediculus humanus corporis*]
					130	22.31	380	500	GI|954303764|gb|KRX92080.1|	Xylosyltransferase sqv-6 [*Trichinella pseudospiralis*]
8	TRAVE DRAFT_ 73853	WSC-domain-containing protein	768bp	GI|636622007|ref|XP_008042138.1|	326	24.54	452	742	GI|701771360|gb|KGQ06159.1|	Xylosyltransferase oxt [*Beauveria bassiana D1-5*]
					313	25.88	315	626	GI|949391510|dbj|GAP85657.1|	putative copper radical oxidase [*Rosellinia necatrix*]
					302	25.5	449	742	GI|908390298|gb|KND90936.1|	Xylosyltransferase oxt [*Tolypocladium ophioglossoides CBS 100239*]
					301	44.52	5	296	GI|949393989|dbj|GAP83249.1|	putative wsc domain protein [*Rosellinia*

										necatrix]
					248	28.63	502	745	GI|949391510|dbj|GAP85657.1|	putative copper radical oxidase [*Rosellinia necatrix*]
					222	27.93	524	742	GI|919012629|ref|XP_013390783.1|	PREDICTED: xylosyltransferase oxt-like [*Lingula anatina*]
					221	24.89	270	481	GI|949391510|dbj|GAP85657.1|	putative copper radical oxidase [*Rosellinia necatrix*]
					219	22.83	381	592	GI|972142722|gb|KUI68971.1|	Xylosyltransferase oxt [*Valsa mali*]
					213	26.76	532	732	GI|972142722|gb|KUI68971.1|	Xylosyltransferase oxt [*Valsa mali*]
					204	26.96	542	742	GI|925201043|gb|JAI52582.1|	putative xylosyltransferase oxt, partial [*Rhodnius neglectus*]
9	TRAVEDRAFT_73850	WSC-domain-containing protein	463bp	GI|636622003|ref|XP_008042136.1|	283	44.17	147	6	GI|949393989|dbj|GAP83249.1|	putative wsc domain protein [*Rosellinia necatrix*]
					176	27.84	112	8	GI|1003743824|gb|KXX79436.1|	Xylosyltransferase oxt [*Madurella mycetomatis*]
					140	27.86	93	4	GI|780631854|ref|XP_011685660.1|	PREDICTED: xylosyltransferase oxt [*Wasmannia auropunctata*]
					137	35.77	81	3	GI|949391510|dbj|GAP85657.1|	putative copper radical oxidase [*Rosellinia necatrix*]
					133	25.56	91	3	GI|908390298|gb|KND90936.1|	Xylosyltransferase oxt [*Tolypocladium ophioglossoides* CBS 100239]
					132	28.79	73	7	GI|961440096|emb|CUT99591.1|	xylosyltransferase [*Echinococcus multilocularis*]
					130	30.77	67	7	GI|972140057|gb|KUI66306.1|	Xylosyltransferase oxt [*Valsa mali*]
					128	28.13	76	3	GI|949391510|dbj|GAP85657.1|	putative copper radical oxidase [*Rosellinia necatrix*]

Table S5. Primers used in this study.

Primer Name	Sequence (5'-3')	Tm	Length(bp)
GAPDH-F	CGGAATCAACGGTTTAGGTC	57.2	221
GAPDH-R	TCTCCTGGAAGACGGTGATG	57.9	
1-GI636624487-F	TCCCGAGCCATTACTACTACG	57.4	197
1-GI636624487-R	GAATCTATGACGCTGATACTGTGC	58.7	
2-GI636624477-F	CTGATGATTCCAACCCCTTTC	58.5	189
2-GI636624477-R	ATACTGAACCGCCAACGATAC	57.0	
3-GI636605689-F	CGGGGAGAAACAACAGTCAT	57.2	193
3-GI636605689-R	ATGGCTCAGGATGAATGGG	57.6	
4-GI636620747-F	TCTGCCCCTTTCCCTTTTC	59.1	124
4-GI636620747-R	GCTTTGATCGCTGCTCTAGTTC	59.0	
5-GI636606177-F	CTGCTTTGACTCGGCAATAGA	58.3	98
5-GI636606177-R	AGAAAACTGGTGAAGGGGAAA	58.1	
6-GI636616829-F	ACCATTCCCCAGCAACTACC	58.8	226
6-GI636616829-R	GGTTCAAGTGGCGTCAGGA	58.8	
